# Exploring biodiversity and ethnobotanical significance of *Solanum* species in Uzbekistan: unveiling the cultural wealth and ethnopharmacological uses

**DOI:** 10.3389/fphar.2023.1287793

**Published:** 2024-01-24

**Authors:** Yusufjon Gafforov, Milena Rašeta, Muhammad Zafar, Trobjon Makhkamov, Manzura Yarasheva, Jia-Jia Chen, Moldir Zhumagul, Mengcen Wang, Soumya Ghosh, Arshad Mehmood Abbasi, Akramjon Yuldashev, Oybek Mamarakhimov, Areej Ahmed Alosaimi, Dilfuza Berdieva, Sylvie Rapior

**Affiliations:** ^1^ Central Asian Center for Development Studies, New Uzbekistan University, Tashkent, Uzbekistan; ^2^ School of Engineering, Central Asian University, Tashkent, Uzbekistan; ^3^ Institute of Botany, Academy of Sciences of Republic of Uzbekistan, Tashkent, Uzbekistan; ^4^ Department of Chemistry, Biochemistry and Environmental Protection, Faculty of Sciences, University of Novi Sad, Novi Sad, Serbia; ^5^ Department of Plant Sciences, Quaid-i-Azam University, Islamabad, Pakistan; ^6^ Department of Forestry and Landscape Design, Tashkent State Agrarian University, Tashkent, Uzbekistan; ^7^ Department of Education and Training Management, Tashkent International University of Education, Tashkent, Uzbekistan; ^8^ College of Landscape Architecture, Jiangsu Vocational College of Agriculture and Forestry, Zhenjiang, China; ^9^ Faculty of Biology and Biotechnology, Al-Farabi Kazakh National University, Almaty, Kazakhstan; ^10^ Higher School of Natural Sciences, Astana International University, Astana, Kazakhstan; ^11^ State Key Laboratory of Rice Biology and Breeding, Ministry of Agricultural and Rural Affairs Laboratory of Molecular Biology of Crop Pathogens and Insects, Zhejiang University, Hangzhou, China; ^12^ Department of Genetics, Faculty of Natural and Agricultural Sciences, University of the Free State, Bloemfontein, South Africa; ^13^ Department of Environmental Sciences, COMSATS University Islamabad, Abbottabad Campus, Abbottabad, Pakistan; ^14^ Department of Ecology and Botany, Andijan State University, Andijan, Uzbekistan; ^15^ Department of Ecology Monitoring, National University of Uzbekistan, Tashkent, Uzbekistan; ^16^ Biology Department, College of Science, Imam Abdulrahman Bin Faisal University, Dammam, Saudi Arabia; ^17^ Department Faculty and Hospital Therapy -1, Occupational Pathology, Tashkent Medical Academy, Tashkent, Uzbekistan; ^18^ Centre d’Ecologie Fonctionnelle et Evolutive, Centre National de Recherche Scientifique, Ecole Pratique des Hautes Etudes, Institut pour la Recherche et le Développement, University of Montpellier, Montpellier, France; ^19^ Laboratory of Botany, Phytochemistry and Mycology, Faculty of Pharmacy, University of Montpellier, Montpellier, France

**Keywords:** Avicenna, biodiversity, Central Asia, ethnomedicine, flowering plant, folk medicine, Solanaceae

## Abstract

Despite its millennial existence and empirical documentation, the ethnological knowledge of herbs is a more recent phenomenon. The knowledge of their historical uses as food, medicine, source of income and small-scale businesses, and the sociological impacts are threatened due to the slow ethnobotanical research drive. Species of the genus *Solanum* have long been extensively used in folk medicine to treat various illnesses of humans since the dawn of civilization. All data were systematically obtained from papers, monographs, and books written in Uzbek, Russian, and English through various scientific online databases, including Google, Google Scholar, PubMed, Scopus, Semantic Scholar, Science Direct, and Web of Science using specific keywords focused on eight *Solanum* species. Eight native and non-native *Solanum* species as *S. dulcamara* L., *S. lycopersicum* L., *S. melongena* L., *S. nigrum* L., *S. rostratum* Dunal., *S. sisymbriifolium* Lam., *S. tuberosum* L., and *S. villosum* Mill. have been recorded in Uzbekistan of Central Asia. In this article we presented recently obtained data on the diversity, morphological characteristics, global distribution, habitat, population status, phenology, reproduction, pharmacology and phytochemistry of these *Solanum* species in Uzbekistan. Furthermore, relying on a combination of literature reviews and analyses from various scientific papers, we focus on food consumption coupled with global ethnobotanical and ethnopharmacological uses in human diseases of the *Solanum* species growing in Uzbekistan. Since the dawn of civilization, these eight cultivated and non-cultivated species of *Solanum* have provided sustainable resources of medicinal plants in Uzbekistan to prevent and treat various human diseases. Based on the collected data, it was shown that *Solanum* species have not been studied ethnobotanically and ethnomedicinally in Uzbekistan and it is necessary to conduct phytochemical and biotechnological research on them in the future. Traditional uses and scientific evaluation of *Solanum* indicate that *S. nigrum*, *S. sisymbriifolium* and *S. tuberosum* are one of the most widely used species in some parts of the world. Although considerable progress has been made to comprehend the chemical and biological properties of *S. nigrum* and *S. tuberosum* species, more research on the pharmacology and toxicology of these species is needed to ensure the safety, efficacy, and quality of their biologically active extracts and isolated bioactive compounds. Additionally, conducting additional research on the structure-activity relationship of certain isolated phytochemicals has the potential to enhance their biological efficacy and advance the scientific utilization of traditional applications of *Solanum* taxa.

## Introduction

Despite its millennial existence and empirical documentation, the ethnological knowledge of herbs is a more recent phenomenon. Knowledge of their historical uses as food, medicine, source of income and small-scale businesses, and the sociological impacts are threatened due to the slow ethnobotanical research drive. The poor documentation and lack of study of medicinal plants in many developing countries has created inconsistencies in their uses in relation to the practice of traditional medicine, food and mythological beliefs. Their relevance in modern-day pharmaceutics and nutraceuticals is a product of human experimentations over time. Factors that may be anthropogenic, ethnographic, and environmental have been implicated in herb underutilization and under-exploration of plants, algae, and fungi, including animals in Central Asia. Ethnobiological literature on Central Asia is scant, random, limited in scope and fraught with taxonomic inconsistencies (Khojimatov et al., 2023a; Gafforov et al., 2023). Hence, this study is based on an extant ethnobotanical treatise and aims to represent an integrative knowledge of the beneficial *Solanum* species of Uzbekistan, their uses in indigent cultures, encompassing a brief phytochemical overview.

With 102 genera and roughly 2,500 species, the flowering plants of Solanaceae (order Solanales), also known as the nightshade or potato family, is very important economically as a source of food and medicine (Särkinen et al., 2018; Kaunda and Zhang, 2019; Chidambaram et al., 2022). *Solanum* with ca. 1,250 species, is the largest genus in the Solanaceae and one of most species-rich genera of flowering plants with contains members spread all over the world, and in temperate zones, there are very few species, while the entire United States and Canada only have roughly 50 species from various genera of Solanaceae (Morris and Taylor, 2017; Gagnon et al., 2022). Its dark colloquial name of “nightshade” comes from the deadly alkaloids found in some family species. South America, where most nightshade species are thought to originate, is home to many of these species. The richest in terms of species diversity are the continents of Africa and Australia. The Solanaceae family is primarily found in tropical and temperate regions, from desert areas to tropical woods. The Solanaceae members have been found on various continents due to their Neotropical origin (Dupin et al., 2016; Tovar et al., 2021). Solanaceae species are used in folk medicine, traditional culture, pharmacology and ornamental gardening. Like the whole world, Uzbekistan also depends heavily on some members of this family as food crops. For instance, food crops produced 540 million tons worldwide in 2010 on 28 million hectares of land. However, this only applies to the four principal crops: potatoes, tomatoes, eggplants, and peppers. It does not apply to many other cultivated species or numerous semi-cultivated, wild-collected species. The main problem of tomato yield in Uzbekistan is the post-harvest activities and due to which a lot of crop production can be wasted (Padalia, 2014). The members are often herbs and can be annuals, biennials, or perennials, while certain species can also be shrubs or small trees.

More than 1,200 wild medicinal plants in Uzbekistan have been studied and described (Khojimatov, 2021). However, many medicinal plants found in Uzbekistan have not been thoroughly scientifically evaluated for their potential value in ethnobotany and ethnomedicine, such as a member of the Solanaceae family. This review aims to investigate the diversity, ethnobotanical uses, and brief details of the phytochemistry and pharmacology of eight *Solanum* species that are cultivated in Uzbekistan, both native and non-native.

## Materials and methods

### The geographic location, vegetation biomes and climate of the study area

**FIGURE 1:**
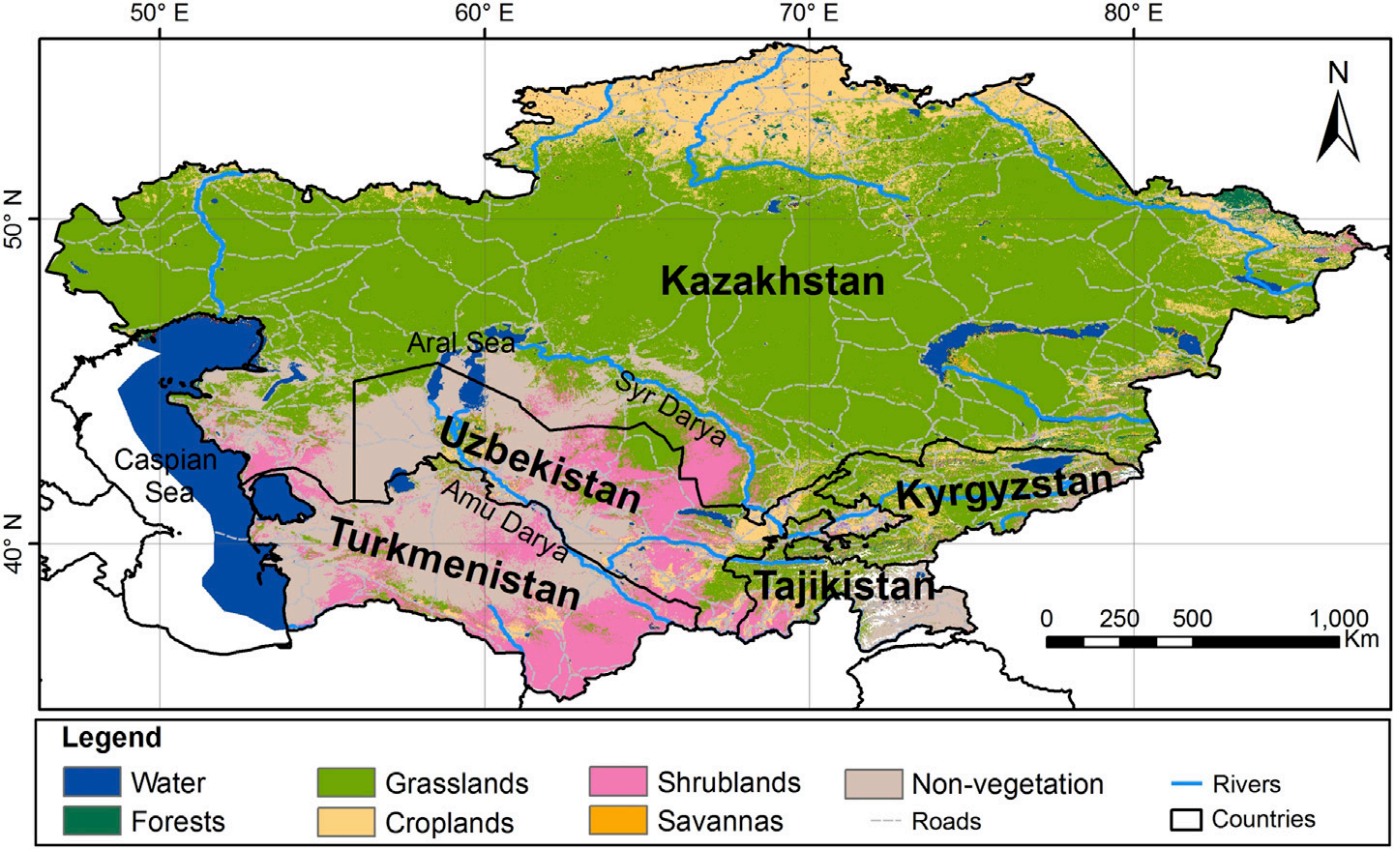
Map of Uzbekistan (Gafforov et al., 2017).

Uzbekistan, located in Central Asia, which covers a total area of 447,400 km^2^ (Figure 1), has a diversity of habitats of global and regional importance for ecological functions.

Uzbekistan’s varied landscapes, consisting of high mountain ranges, vast steppes, deserts and riparian wetlands result in a high diversity of habitats. The mountains of the Central Asia biodiversity region within the largest floral geographic region of temperate Asia consist of two major mountain systems, the Pamir and the Tien Shan. The mountainous areas occupy 15% of the territory of Uzbekistan. The highest point in Uzbekistan is the peak of Hazrati Sultan in the Hissar mountain range (4,643 m, 15,233 ft.) in the Surkhandarya region in Southern Uzbekistan (Gafforov, 2017).

**FIGURE 2:**
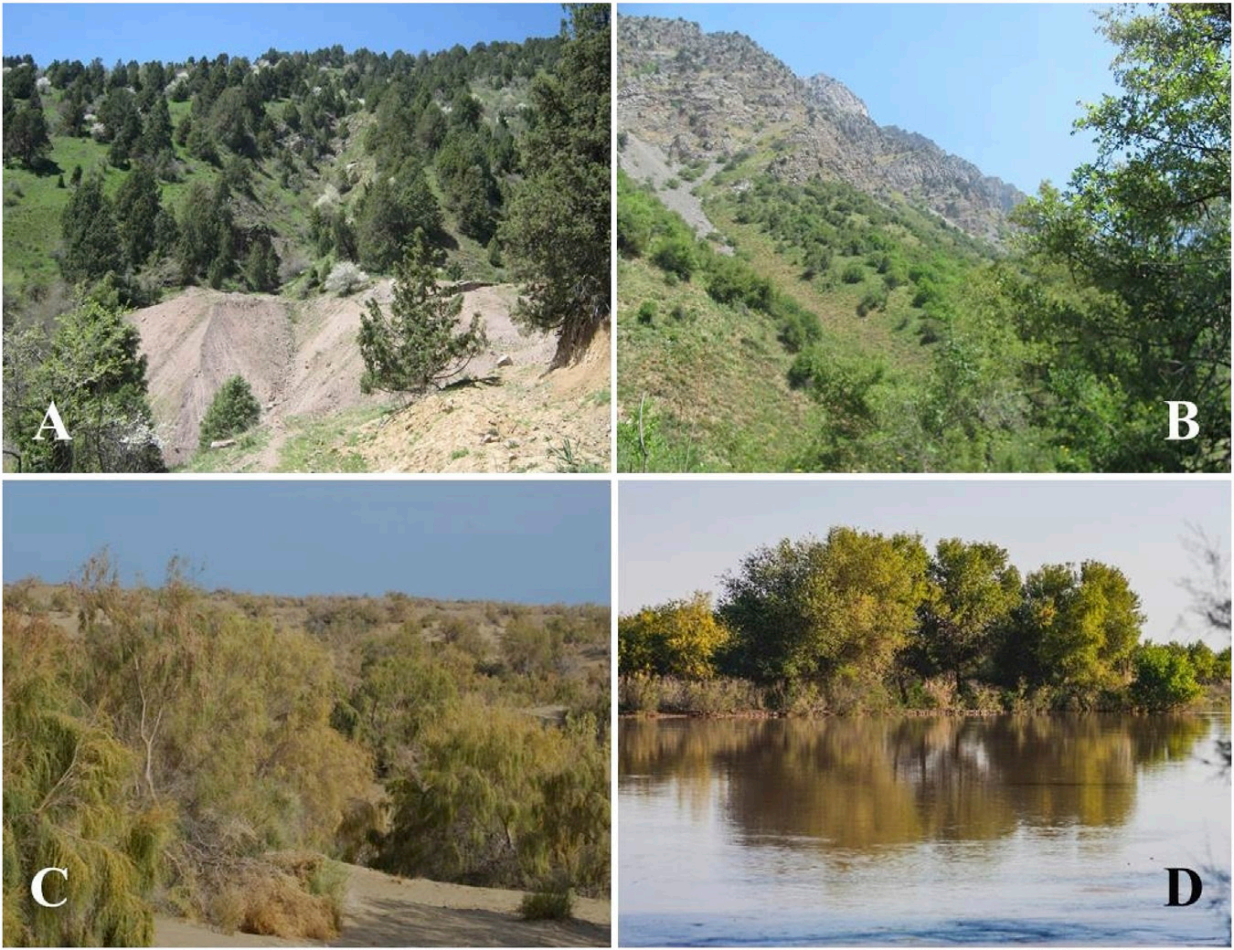
Forest types in the study area. **(A)** Mountain juniper forests; **(B)** Wild fruit tree forests in the mountain; **(C)** Desert saxaul (*Haloxylon* spp.) forests; **(D)** Tugai forests (Gafforov et al., 2020).

The largest biomes in Uzbekistan are temperate grasslands, savannas, and shrublands. Uzbekistan also contains mountain grasslands and shrublands, deserts, and xeric shrublands, as well as temperate coniferous forests biomes. Despite the mountainous nature of Central Asia, forests cover a relatively small proportion of each country. Much of the forest area is dominated by small trees of the genus *Haloxylon* Bunge ex E. Fenzl (Amaranthaceae) and other shrubs, particularly in desert and semi-desert areas of Uzbekistan. In moist, mountainous areas the main species are *Juniperus* spp., *Populus* spp., *Salix* spp., *Juglans regia* L., *Pistacia vera* L., *Malus sieversii* (Ledeb.) M. Roem., and *M. niedzwetzkyana* Dieck ex Koehne, *Prunus communis* L., *P. sogdiana* Vassilcz., *P. ferganica* Lincz, *Pyrus bucharica* Litv., and *P. korschinskyi* Litv., *Sorbus persica* Hedl., and other deciduous forest trees, fruit-bearing trees, and shrubs (Botman, 2009; Gafforov et al., 2017). The flora of Uzbekistan includes 4,500 species of vascular plants, of which about 400 species are endemic, rare, and relict and about 200 species are used in foods, and 1,200 species are used as medicinal plants (Khojimatov et al., 2023a,b). According to the World Geographical Scheme for Recording Plant Distribution system, the region of Uzbekistan belongs to the Central Asian botanical flora (Brummit, 2001). The main ecological forest types in Uzbekistan are mountain, desert, and flood-plain forests (Figure 2).

Uzbekistan is one of the major producers of fruits and vegetables among the Commonwealth of Independent States (CIS) nations due to fertile land. Many farms focus on growing specific crop species, such as potatoes, tomatoes, peppers, melons, and watermelons, in order to maximize productivity and profitability. In both open fields and greenhouses, tomatoes are the most popular vegetable crop in Uzbekistan. Fresh tomatoes are a profitable crop that can boost the profitability of greenhouse farmers because 20% of their production in open fields and roughly 60% in protected regions are exported.

### Population of Uzbekistan

Nowadays, the population of Uzbekistan is more than 35,163,944 people (Macrotrends, 2023). Many nationalities and ethnic groups, such as Uzbeks, make up more than four-fifths of the population, followed by Tajiks, Kazakhs, Tatars, Russians, Karakalpaks and other Germans, Greeks, Kyrgyz, Meskhetian Turks, Slavs Turkmens, Uighurs, and Ukrainians. In addition, numerous Diasporas in Uzbekistan are Armenians, Azerbaijanis, Georgians, Iranians, Koreans, and many other nationalities (Lubin, 1984).

### Data collection

We have obtained all data from papers, monographs, and books written in Uzbek, Russian, and English in indexed and non-indexed journals by using online bibliographic databases: Google, Google Scholar, PubMed, Scopus, Semantic Scholar, Web of Science, and ScienceDirect Navigator, as well as some local library sources, and other available scientific materials, focused on eight *Solanum* species. As a result, approximately 270 published articles were found in which some studies were selected for the diversity, geographical distribution, habitat, taxonomy, morphological characteristics, ethnobotany, and uses in ethnomedicinal of the selected plants of the genus *Solanum*. Moreover, we investigated the reference lists of 190 selected literature sources from the year range 1930–2023 to acquire a more comprehensive and precise dataset of information. In addition, the scientific names of the plants were checked for potential synonyms in Plants of the World Online (POWO) (Powo, 2023), and a current list of *Solanum* species was compiled as well.

## Results and discussion

### Diversity of *Solanum* species in Uzbekistan

The largest genus of Solanaceae, *Solanum* L., has over 1,250 species, making it economically and culturally significant for its food crops (Kaunda and Zhang, 2019; Gagnon et al., 2022). This perennial, frost-sensitive shrub needs bright, humid weather. This genus has spread throughout the Old World, including Australia, Africa, as well as North and South America, Europe, and Asia. Its main producers are India, Pakistan, Sri Lanka, Bangladesh, China, Japan, Uzbekistan, and Syria (Devaux et al., 2021). Uzbekistan grows along agricultural lands, built-up regions, roadside ditches, lowland river basins, and disturbed places. Based on the Plants of the World Online database and recently published articles in Uzbekistan, the scientific names of eight species of the genus *Solanum* are listed: *Solanum dulcamara* L., *S. lycopersicum* L., *S. melongena* L., *S. nigrum* L., *S. rostratum* Dunal., *S. sisymbriifolium* Lam., *S. tuberosum* L., and *Solanum villosum* Mill. *Solanum* encompasses a limited number of species (Table 1).

**TABLE 1:** List of the eight *Solanum* species in Uzbekistan.

Plant name	Local name	English name	Plant type	Edible part
*Solanum dulcamara*	Nordon ituzum	Bittersweet, Bittersweet nightshade, Bitter nightshade, Blue bindweed	Scandent subshrub	—
*Solanum lycopersicum*	Pomidor, Pomildori	Tomato, Tomatoes	Annual	Fruit/vegetable
*Solanum melongena*	Baqlajon	Eggplant, Aubergine	Annual	Fruit
*Solanum nigrum*	Qora ituzum, Qora mevali ituzum	Black nightshade, Blackberry nightshade	Annual	Fruit
*Solanum rostratum*	Tikanli ituzum, Tumshuqsimon ituzum	Buffalobur nightshade, Buffalo-bur, Spiny nightshade, Colorado bur, Kansas thistle, Mexican thistle	Annual Seed propagated	—
*Solanum sisymbriifolium*	Qurttanabarg ituzum	Vila-vila, Sticky nightshade, Red buffalo-bur, Morelle de Balbis	Annual	Fruit
*Solanum tuberosum*	Kartoshka	Potato	Perennial herb	Tubers
*Solanum villosum*	Qizil ituzum, Yumshoq tukli ituzum	Hairy nightshade, Red nightshade, Woolly nightshade	Annual	Leafs

Food category	Application	Origin status	Distribution	Vegetation zone/ecosystems
Wild plants	Use as a medicine	Native	Native to Europe and some parts of temperate Asia, alien in many temperate regions of the world	Temperate zone
Cultivated vegetable	Fruits are used as a vegetable	Alien, cultivated, sometimes escaped (ephemerophyte)	Native to Peru and adjacent regions, cultivated elsewhere	Sub-tropical to temperate zone
Cultivated vegetable	Leaves have narcotic properties and the seeds are used as a stimulant	Alien, cultivated, not escaped	Native to South Asia, naturalized	Tropical to temperate zone
Wild fruit	Fresh fruit used	Native	Native to temperate Eurasia, alien in North America and Australia	Temperate zone
Wild plant	Infusion of the powdered root, taken for a sick stomach	Alien, unintentionally introduced	Native to North and Central America, alien in the arid regions of the world	Tropical to temperate regions wide distribution
Wild fruit	Use as a medicine, and fruits can be eaten as boiling or as a raw fruits	Alien unintentionally introduced	Native to South America, alien in many parts of the world	Tropical to subtropical zone
Cultivated vegetable	The fresh part is cooked and used as a vegetable	Alien, cultivated, not escaped	Native to the mountainous areas of Mexico in North America and Chile, Peru in South America	Temperate zone
Cultivated vegetable	Fresh and dry consumed as a vegetable	Alien, unintentionally introduced	Afghanistan, India, Nepal; South western Asia, Europe	Tropical-subtropical zone

Several species, particularly *Solanum nigrum* and *S. dulcamara,* are considered nightshades and highly poisonous. The potato (*S. tuberosum*), tomato (*S. lycopersicum*), and eggplant (*S. melongena*) are three food crops of significant economic importance that belong to the wide and diversified genus *Solanum* of flowering plants (aborigine, brinjal). It also includes various plants grown for their decorative blooms and fruits and the so-called horse nettles, which are unrelated to the *Urtica* genus of real nettles. *Solanum* species have many different growth habits, including annuals, perennials, vines, subshrubs, shrubs, and tiny trees. Many once separate genera, like *Lycopersicon* (the tomato group) and *Cyphomandra,* are now subgenera or sections of *Solanum*. Of the eight selected species, only two are native: a sub shrubby climbing *S. dulcamara* and an annual *S. nigrum.* All others are invasive: *S. lycopersicum*, *S. melongena* and *S. tuberosum* are widely cultivated, and three other species, i.e., *S. villosum* from the Mediterranean area, while *S. rostratum* and *S. sisymbriifolium* from North and South America, respectively are unintentionally introduced species that can be classified as the least naturalized species in the lowland dump places of the country.

Fresh fruit yields of 45–65 tons/ha under irrigation, of which 85 to 95 percent is moisture, are considered to be good commercial yields. For the purposes of this investigation, a 15% dry matter content was assumed. FAOSTAT estimates that Uzbekistan’s yields are higher than the global average but lower than those of the major Mediterranean Sea producing nations (Spain, Italy, etc.). Fresh yields in Uzbekistan typically range between 25 and 35 tons per hectare, according to both FAOSTAT and municipal figures.

### Economic importance of the *Solanum* in Uzbekistan

Uzbekistan has reforming its economic and agricultural policies and given priority to the development of the horticultural subsector. Uzbekistan is well known for its delicious fruits and vegetables; with its entrepreneurial dynamics, it has enormous potential to become a key player in the production and export of horticultural products as well as value-added food products. Among them economically important potato and tomato crops are planted in Uzbekistan and a high yield is obtained from them every year. According Ministry of Agriculture of Uzbekistan in January-December 2022, 301,000 tons of agricultural products were grown in greenhouses (East-Fruit, 2022; Future Food Production, 2022). Of these, 211 thousand tons of tomatoes were harvested. Potatoes for the 2022 harvest in all categories of farms in Uzbekistan are planted on 243,000 ha, which is 55%, 86,000 ha more than last year. Accordingly, the forecasted crop volume in 2022 was 4.2 million tons, which is 26% as 850,000 tons more than in 2021. However, many *Solanum* cultivars have lost their yield due to damage caused by various pathogens. As a result, it causes great loses to the economy of the state (Gafforov et al., 2022).

### Botanical traits and distribution, taxonomy, habitat, ecology, phytochemistry and pharmacology

*Solanum* is one of the largest genus of flowering plants (Frodin, 2004), well known for the Black Nightshade, *Solanum nigrum* (Solanaceae). The botanical traits of *S. dulcamara*, *S. lycopersicum*, *S. melongena*, *S. nigrum*, *S. rostratum*, *S. sisymbriifolium*, *S. tuberosum*, and *S. villosum* are as follows.

### Taxonomic treatment

**FIGURE 3:**
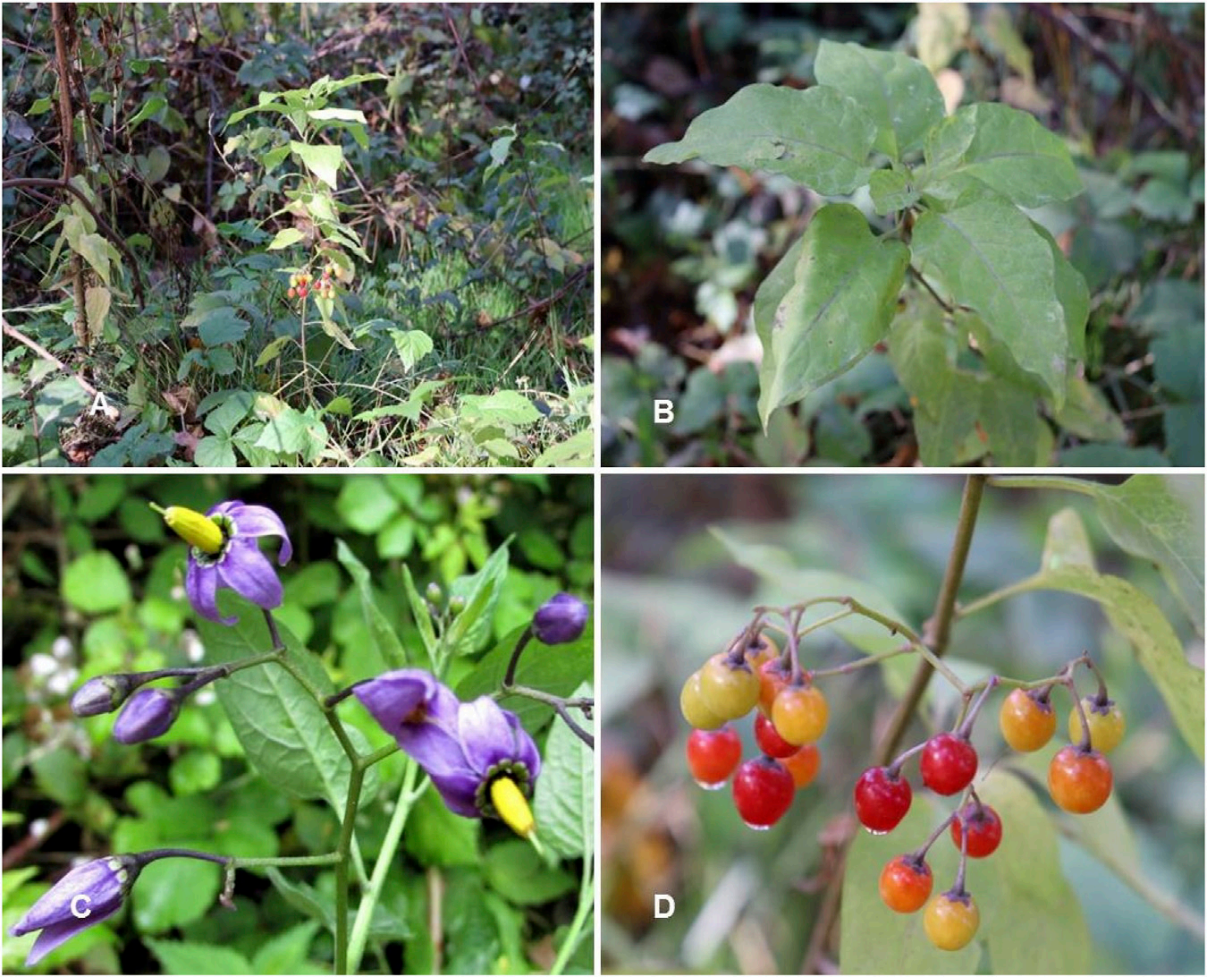
*Solanum dulcamara*. **(A,B)** Habit and leaves; **(C)** Inflorescence; **(D)** Fully mature fruits (Tashkent Botanical Garden: 17.11.2022, Photo credit by Yusufjon Gafforov and Trobjon Makhkamov).

*Solanum dulcamara* L. Sp. Pl.: 185 (1753) (Table 1; Figure 3)

Synonyms: *Dulcamara flexuosa* Moench, *Lycopersicon dulcamara* (L.) Medik, *Solanum ruderale* Salisb., nom. superfl., *S. scandens* Neck., nom. superfl.

Description: Unarmed or pubescent semi-shrub with a few upright, ascending, or occasionally climbing shoots coming from the base. (5)7–10 cm long, (2.5)4–5 cm wide, broadly ovate, with a distinct unequal or slightly kidney-shaped, less frequently wedge-shaped base, noticeably attenuated towards the apex, decreasing up the stem, and 2–3 times shorter than the blade on the petioles. Leaves can be whole or have upper leaves incised into two obtuse, ovate, horizontally or upwardly directed lobes. Inflorescence flat corymbose panicle with 10–25 flowers and 1–2 branches at the base. Peduncles that are 3–4.5 cm long and pedicels that are heavily pubescent or nearly glabrous. Calyx (1.5–), 2 (–2.5) mm long, three lobes, and pubescent or hairless. Corolla purple, 15–17 mm in diameter, with 3–4 mm broad, especially in the bud, oblong-ovate lobes, and fluffy at the top. Embryos merged. Red, round, (6) 7–8 mm in diameter berries are present (Solanum dulcamara, 2023).

Phenology: Flowers in June-September and fruits in July-September.

Reproduction: By seeds.

Population status: Common, forming dense groups.

Global Distribution: *Solanum dulcamara* is a native plant of several African countries (Algeria, Morocco, and Tunisia). Asia (Afghanistan, China, Inner Mongolia, Iran, Iraq, Japan, Kazakhstan, Khabarovsk, Kirgizstan, Mongolia, Myanmar, Pakistan, Palestine, Tajikistan, Turkmenistan, Uzbekistan, Vietnam). Europe (Albania, Austria, Belarus, Belgium, Bulgaria, Denmark, Finland, France, Germany, Greece, Hungary, Ireland, Italy, Netherlands, Norway, Poland, Portugal, Romania, Russia, Serbia, Slovakia, Spain, Sweden, Switzerland, Turkey, Ukraine, United Kingdom). It is introduced to North America (Canada, United States) and South America (Brazil).

Habitat: *Solanum dulcamara* grows in roadsides, disturbed grounds, mountain river valleys and lakesides, lowland river valleys and sides of irrigation canals, built-up areas, and agricultural lands.

Phytochemistry and Pharmacology: The chemical composition of different parts of *S. dulcamara* was discussed especially in terms of alkaloid identification, bioactivity and isolation. Cansever and Turker (2007) found in their research that methanolic extracts derived from leaves and stems of *S. dulcamara* grown in natural field conditions demonstrated effective antibacterial properties against *Staphylococcus epidermidis*, *S. aureus*, *Klebsiella pneumonia*, *Salmonella typhimurium*, and *Serratia marcescens*. Notably, the antibacterial efficacy was higher in field-grown plant material compared to in vitro-grown material. Furthermore, the methanolic extracts exhibited superior antitumor activity compared to water extracts, with field-grown leaves and stems displaying greater efficacy than their *in vitro*-grown counterparts (Cansever and Turker, 2007). Two years later, Kumar et al. (2009), reported that the plant produces a high content of a specific alkaloid: β-solamarin (roots), solanine (unripe fruits) and solasodine (flowers). All parts of the plant contain various glycoalkaloids of a wide structural variety (solamarin, solamargine, solanine, solasodine, tomatidine), phenolic compounds (biflavonoids) and steroids (β-sitosterol and stigmasterol) (Rosa-Martínez et al., 2015; Popova et al., 2021). Additionally, Rosa-Martínez et al. (2015) isolated and examined flavonoids from *S. dulcamara* to investigate their potential anti-hyperglycemic properties.

**FIGURE 4:**
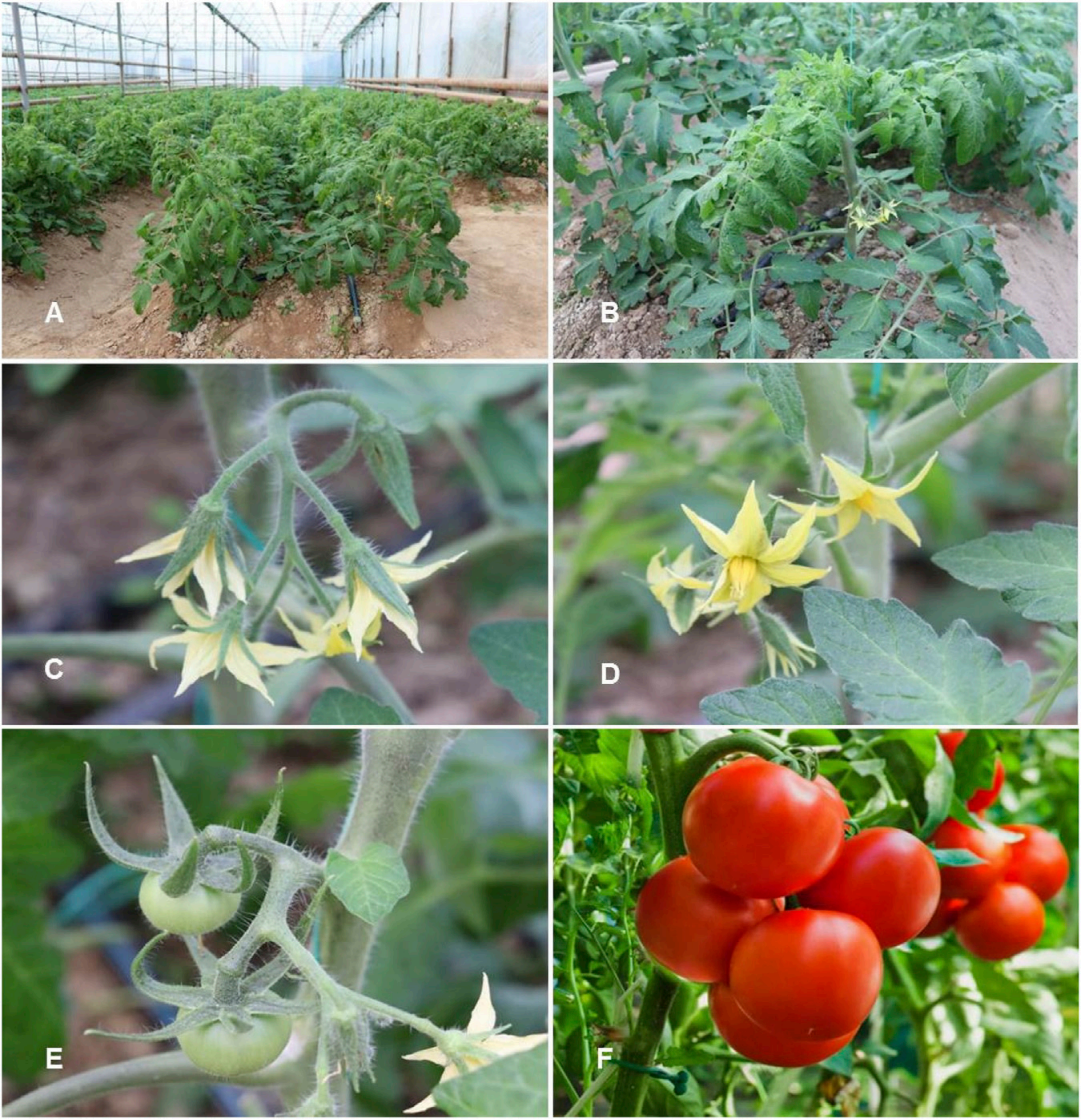
*Solanum lycopersicum.* **(A,B)** Habit; **(C,D)** Inflorescence in hairy individual; **(E)** Immature infructescence; **(F)** Fully mature fruits (Tashkent province, Qibray district, TashGres, Greenhouse, 28.12.2022, Photo credit by Yusufjon Gafforov and Trobjon Makhkamov).

*Solanum lycopersicum* L. Sp. Pl.: 185 (1753) (Table 1; Figure 4)

Synonyms: *Lycopersicon esculentum* Mill., *L. esculentum* subsp. *typicum* Luckwill, not validly publ., *L. lycopersicum* (L.) H. Karst., nom. rej., *L. pomum-amoris* Moench, nom. superfl., *L. solanum-lycopersicum* Hill, nom. superfl., *Solanum lycopersicum* var. *esculentum* (Mill.) Voss.

Description: The two distinguishing features of *Solanum lycopersicum* are its little, soapy-smelling, green fruits with a disagreeable flavor and its small compound leaves with thick, rounded leaflets. Leaflets are disseminated at the edge blastozone and go through developmental stages like leaves. The tomato is a perennial plant in the Solanaceae, frequently grown as an annual herb. It usually reaches a height of 1–3 m and has a flimsy woody stem that scrambles over neighboring plants. Tomatoes can be oblong, round, flat on top and bottom, or pear-shaped. The fruit is a tasty, brightly colored, frequently red berry that is typically much larger in cultivated varieties than it is in wild plants. This is because of the pigment lycopene (Solanum lycopersicum, 2023).

Phenology: The time of flowering and fruiting depends on early or late varieties of tomato and it depends on which region of Uzbekistan it is planted.

Reproduction: By seeds.

Population status: Common/Cultivated.

Global distribution: *S. lycopersicum* is native to South America but was introduced into countries of Asia, Europe and North America, where they soon became popular and were exported around the world. There are no naturally growing wild species of tomato in Uzbekistan.

Habitat: Not known in a truly wild situation. Wild tomatoes are native to western South America and distributed from Ecuador to northern Chile (Darwin et al., 2003; Peralta and Spooner, 2005). They flourish in a range of environments, including those with arid coastal lowlands and surrounding regions where the pacific winds are scarcer in the fall and wet climates, isolated valleys in the high Andes, and harsh deserts as the Atacama Desert in northern Chile.

Phytochemistry and Pharmacology: The chemical composition of *S. lycopersicum* covers a broad spectrum of bioactive coumpouds and is very well documented, based mainly on phenolic compounds, carotenoids and alkaloids as the most present (Helmja et al., 2007; Iijima et al., 2009; Choi et al., 2011; Hövelmann et al., 2019). Choi et al. (2011) determined phenolic compounds viz. caffeic acid-hexose isomer I, caffeic acid-hexose isomer II, caffeoyl-quinic acid, 5-caffeoylquinic acid, caffeoyl-quinic acid isomer, quercetin-3-apiosylrutinoside, quercetin-3-rutinoside, dicaffeoylquinic acid, tricaffeoylquinic acid, naringenin chalcone, and naringenin in different varieties of *S. lycopersicum*. According to Elizalde-Romero et al. (2021), *S. lycopersicum* is an important source of lycopene, whereas different preparation of the plant (tangerine tomato juice, red tomato juice, tomato paste, fresh tomato, and tangerine sauce) contained lycopene in both forms, as *cis-*, and *trans-*lycopene. All parts of this plant, including fruits, leaves, and stems, contain steroidal glycoalkaloids as α-tomatine and dehydrotomatine (Ostreikova et al., 2022).

Free amino acid and phenolic derivatives were investigated for antioxidative and cytotoxic properties (Choi et al., 2011). Rosa-Martínez et al. (2015) reported that *S. lycopersicum* is the primary food source of lycopene, a significant source of vitamin C and vitamin E as well as of both flavonoids naringenin and rutin with antioxidant properties. Recent findings also identify a clear connection between tomato and positive effects on metabolic syndrome as hypertension and cardiovascular disease (Alam et al., 2019; Ani et al., 2022). Indeed, Ani et al. (2022) showed a possible mechanism of antihypertensive property of lycopene-rich extract of *Solanum lycopersicon* in Wistar rats.

**FIGURE 5:**
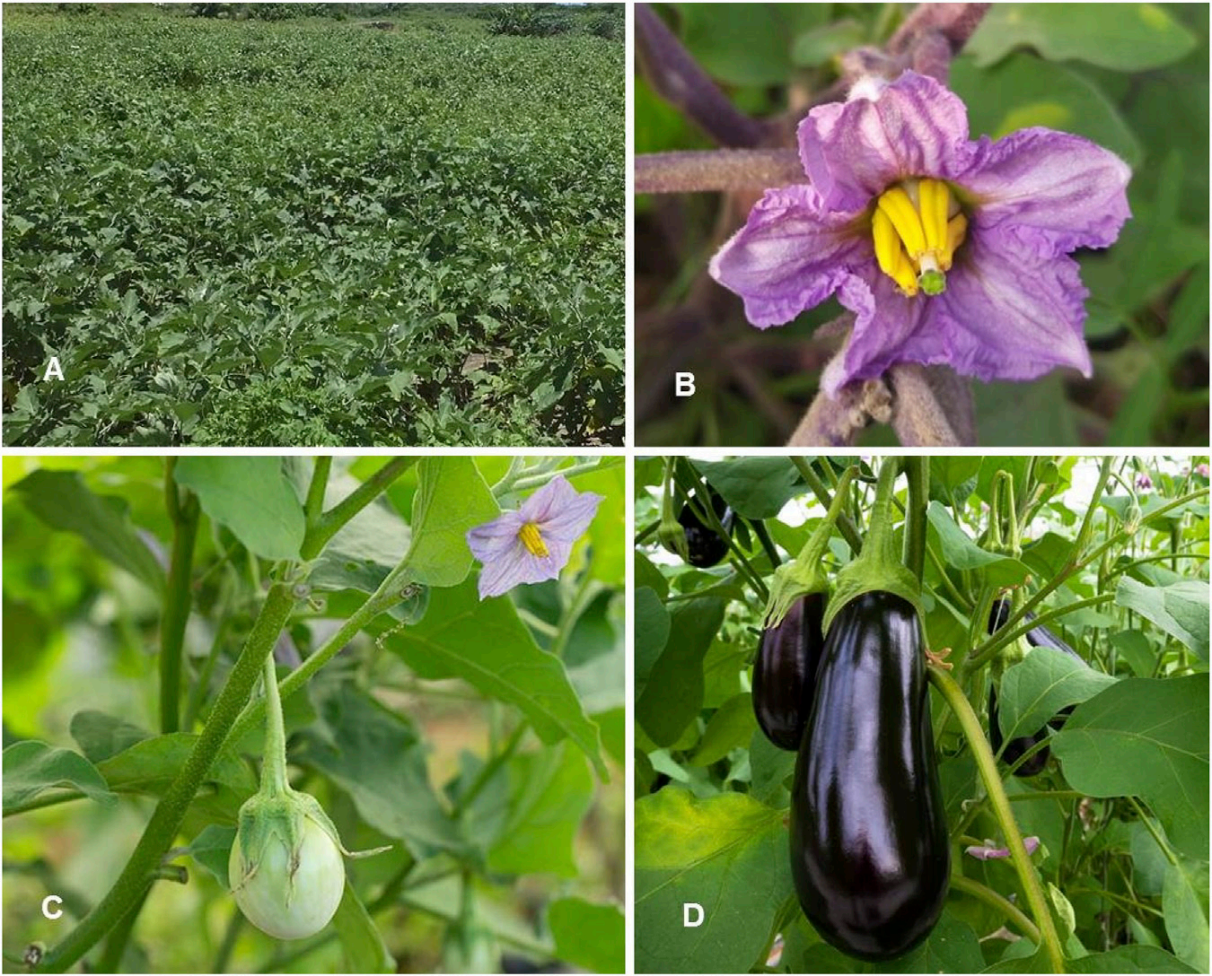
*Solanum melongena*. **(A)** Habit; **(B)** Inflorescence; **(C)** immature infructescence; **(D)** Fully mature fruits (Tashkent province, Qibray district, eggplant farmer area). Photo credit by Yusufjon Gafforov and Trobjon Makhkamov).

*Solanum melongena* L. Sp. Pl.: 186 (1753) (Table 1; Figure 5)

Synonyms: *Melongena esculenta* (Dunal) Grecescu, *M. incurva* Mill., *M. ovata* Mill.,

*M. spinosa* Mill., *M. teres* Mill., *Solanum aethiopicum* var. *violaceum* Dunal, *S. album* Lour., *S. album* Noronha, not validly publ., *S. album* var. *richardii* Dunal, *S. album* var. *rumphii* Dunal, *S. edule* Schumach. & Thonn., *S. edule* var. *multifidum* Dunal, *S. esculentum* Dunal, *S. esculentum* var. *aculeatum* Dunal, *Solanum esculentum* var. *subinerme* Dunal, *S. heteracanthum* Dunal, *S. indicum* Roxb., nom. illeg., *S. lagenarium* Dunal, *S. melongena* subsp. *agreste* Dikii, *S. melongena* var. *angustum* Dikii, *S. melongena* var. *cylindricum* Dikii, *S. melongena* var. *esculentum* (Dunal) Walp., *S. melongena* var. *giganteum* (Alef.) Dikii, *S. melongena* var. *globosi* Dikii, *S. melongena* var. *leucoum* (Alef.) Dikii, *S. melongena* var. *ovigera* Pers., *S. melongena* var. *racemiflorum* Dikii, *S. melongena* var. *racemosum* Dikii, *S. melongena* var. *serpentinum* L.H. Bailey, *S. melongena* var. *stenoleucum* (Alef.) Dikii, *S. melongena* var. *variegatum* (Alef.) Dikii, *S. melongena* var. *violaceum* (Alef.) Dikii, *S. melongena* var. *viride* Dikii, *S. melongenum* St.-Lag., *S. oviferum* Salisb., *S. ovigerum* Dunal, *S. ovigerum* var. *album* Sweet, *S. ovigerum* var. *insanum* Blume, *S. ovigerum* var. *luteum* Sweet, *S. ovigerum* var. *oblongocylindricum* Dunal, nom. superfl., *S. ovigerum* var. *ovum-album* Dunal, *S. ovigerum* var. *ovum-luteum* Dunal, *S. ovigerum* var. *ovum-rubens* Dunal, *S. ovigerum* var. *ruber* Sweet, *S. ovigerum* subsp. *sinuatorepandum* Dunal, *S. ovigerum* subsp. *subrepandum* Dunal, *S. ovigerum* var. *violaceum* Sweet, *S. plumieri* Dunal, *S. pressum* Dunal, *S. pseudoundatum* Blume, *S. pseudoundatum* var. *albiflorum* Blume, *S. pseudoundatum* var. *atropurpurascens* Blume, *S. pseudoundatum* var. *leucocarpon* Blume, *S. requienii* Dunal, *S. sativum* Dunal, *S. sativum* var. *albiflorum* (Blume) Dunal, *S. sativum* var. *atropurpurascens* (Blume) Dunal, *S. sativum* var. *leucocarpon* (Blume) Dunal, *S. serpentinum* Noronha, *S. tomentosum* Hasselt ex Miq., not validly publ., *S. trilobatum* Noronha, not validly publ., *S. trongum* Poir., *S. trongum* var. *divaricatum* Dunal, *S. trongum* var. *rumphii* Dunal, *S. violaceum* DC. ex Dunal, not validly publ., *S. zeylanicum* Scop.

Description: Annual succulent up to 90 cm tall. Few prickles and stellate hairs on the stem and branches. Oval to rhomboid-ovate, sinuate to lobed leaves, 5–20 × 4–15 cm. Purple to pale violet, solitary or in clusters of up to five, recurved pedicel, up to 5 cm long. Campanulate, sparsely prickled, 15–18 mm long calyx that enlarges in fruit. The lobes of the corolla’s limb are triangular-ovate and stellate-tomentose on the outside. The thread of stamen supporting the filament is 3–4 mm long filaments. Berry, 8–15 cm long, ovoid to subglobose to elongated in shape, typically dark purple or different color variants. Three mm long, highly rugose subreniform seeds (Solanum melongena, 2023).

Phenology: The time of flowering and fruiting depends on early or late varieties of eggplants and it varies on which region of Uzbekistan it is planted.

Reproduction: By seeds, propagation by rooting healthy shoots is also possible.

Population status: Common/Cultivated.

Global distribution: *S. melongena* is native to Southeast Asia (China South-Central, Laos, Malaya, Myanmar, Thailand, and Vietnam) and has been cultivated in southern and eastern Asia regions since prehistory for food purposes.

Habitat: Cultivated *S. melongena* cultivars are planted on agricultural lands.

Phytochemistry and Pharmacology: The phytochemical constituents of *S. melongena* are different alkaloids as amides and glycoalkaloids (pyrrolidine, quinazolizidine and tropane), phenolic acids, phenylpropanoids, polyphenols (anthocyanins, flavonoids), steroidal saponins, sterols and tetracyclic triterpenes (Rosa-Martínez et al., 2015; Sun et al., 2015; Lelario et al., 2019; Chen et al., 2021; Ralte et al., 2021; Jit et al., 2022). Kacjan Maršić et al. (2014) reported that the main phenolic compounds of *S. melongena* were chlorogenic acid, delphinidin-3-rutinoside, quercetin-3-glucoside and quercetin-3-galactoside. Sun et al. (2014) isolated ten polycyclic aromatic lignanamides from *S. melongena* roots, including four new melongenamides. Then Sun et al. (2015) characterized 16 phenylpropanoid amides, and among them four compounds (*N-cis-*feruloylnoradrenline, *N*-*trans*-sinapoyloctopamine, *N-trans*-caffeoyloctopamine, and *N-trans*-feruloylnoradrenline) were isolated from the genus *Solanum* for the first time. Yang et al. (2018) isolated and characterized six steroidal saponins, including five new cholestane saponins (abutilosides P-T), one new steroidal alkaloid (abutiloside U), along with one new natural product named as (25*R*)-3β,16α,26-trihydroxy-5-en-cholestan-22-one-3-*O*-α-L-rhamnopyranosyl-(1 → 4)-β-D-glucopyranoside, abutiloside P, and three know steroids (abutiloside G, solaviaside B, and tumacone).

Antiinflammatory lignanamides exhibited inhibition of nitric oxide production in lipopolysaccharide-induced RAW 264.7 macrophages (Sun et al., 2014). Anticholinesterase and antioxidant activities of glycoalkaloids were discussed by Lelario et al. (2019). Jit et al. (2022) demonstrated that *S. melongena* contains two phenolic compounds (chlorogenic acid, ferulic acid) with radioprotective activity.

**FIGURE 6:**
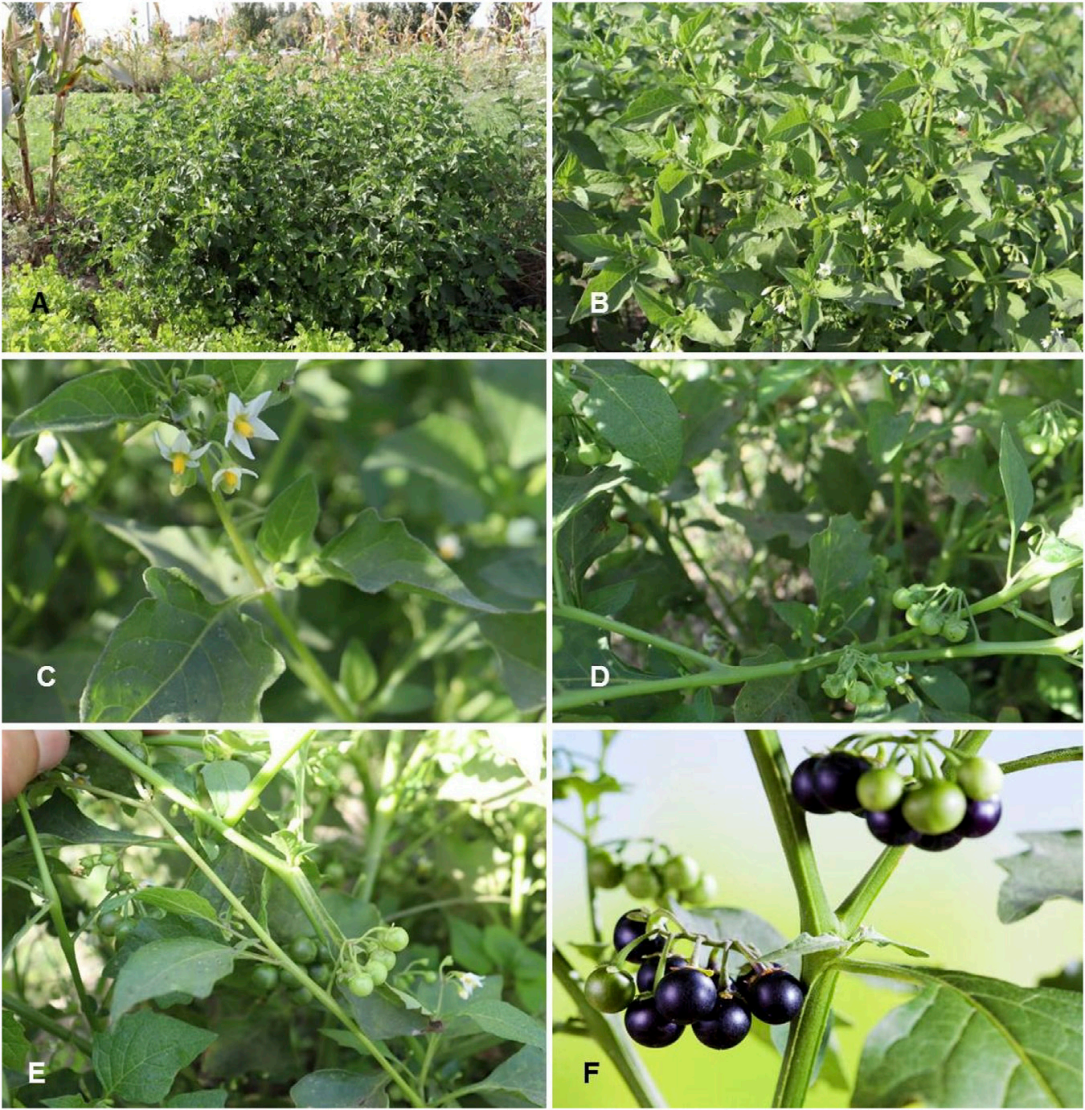
*Solanum nigrum*. **(A,B)** Habit; **(C,D)** Inflorescence and immature infructescence; **(E,F)** Fully mature fruits. (Tashkent province, Pskent district, 29.09.2022, Photo credit by Yusufjon Gafforov and Trobjon Makhkamov).

*Solanum nigrum* L. Sp. Pl.: 186 (1753) (Table 1; Figure 6)

Synonyms: *Solanum humile* Salisb., nom. superfl., *S. morella* Desf, nom. superfl., *S. morella* subsp. *nigrum* (L.) Rouy, nom. superfl., *S. nigrum* var. *genuinum* Hassl., not validly publ., *S. nigrum* var. *humile* Macloskie, not validly publ., *S. nigrum* var. *legitimum* Neilr., not validly publ., *S. vulgatum* Baumg., nom. superfl., *S. vulgatum* var. *nigrum* (L.) Spenn., nom. superfl.

Description: It is an annual plant having a stem that is upright, decumbent, splayed-branched from the base, cylindrical below, flattened-cylindrical, with slightly projecting non-serrated ribs, and 25–50 (75) cm high. The leaves are 3–5–7 cm long, 2–4 cm wide, shorter than blades on petioles, dark green, juicy, glabrous or, especially young, with sparse short hairs, denser along the veins, oblong-ovate or rhombic-ovate, gradually narrowing from the middle into an acute apex, with an unequal wedge-shaped base widely descending onto the petiole, at the bottom notched-toothed. The extra-axillary inflorescences are umbellate, or racemose corymbose with 3–8 flowers each. Pedicels with the same, but more thick hairs, drooping, spaced at fruits; peduncles glabrous or, more frequently, with upward-directed adpressed hairs, and 2–2.5 mm long, cylindrically campanulate, glabrous or more frequently hairy, and one-third incised into blunt teeth. Corolla is white, 6–7 mm in diameter, and has lobes on the outer that are ovate-lanceolate and quickly pubescent. The berry is 6–10 mm long, black, and round. Yellow, almost reniform, somewhat elongated at one end, fine-meshed seeds measure 2 mm in length (Solanum nigrum, 2023).

Phenology: Flowers in June-October, fruits in July-August.

Reproduction: By seeds.

Population status: Common, sometimes forming dense groups in croplands.

Global Distribution: This species occurs on all continents except Antarctica; it is species native to Eurasia (Western Europe to Japan), northern Africa, and Australia, sporadically introduced in South Africa and naturalized locally in temperate North America (Särkinen et al., 2018).

Habitat: Disturbed grounds, mountain river valleys and lakesides, lowland river valleys and sides of irrigation canals, dry riverbeds, alluvial fans and gravel deposits, built-up areas, and agricultural lands.

Phytochemistry and Pharmacology: Jain et al. (2011) reported major bioactive compounds in *S. nigrum* that include glycoalkaloids, glycoproteins, and polysaccharides. The glycoalkaloids include solamargine, solanine, solasonine (Jain et al., 2011; Sivakumar et al., 2020). In 2022, Chen et al. (2022) claimed that about 188 phytochemicals were separated and identified from *S. nigrum*, containing alkaloids, flavonoids, organic acids, phenylpropanoids and their glycosides, polysaccharides and steroids.

Using streptozotocin-induced diabetic mice model, Nyaga et al. (2019) showed significant antidiabetic effect of *S. nigrum* var. *sarrachoides* and hence their use in folklore medicine. Chen et al. (2022) also mentioned that *S. nigrum* has nutrients essential for humans and of great importance to human eye and skin health (α-carotene, β-carotene, ferulic acid ester). Steroidal saponins and steroidal alkaloids have been considered as the main bioactive components of *S. nigrum*, exhibiting various biological activities (Li et al., 2023). Then Wang et al. (2023) proved that the steroidal saponins from *S. nigrum* had broad-spectrum cytotoxic activity against various human leukemia cancer cell lines.

**FIGURE 7:**
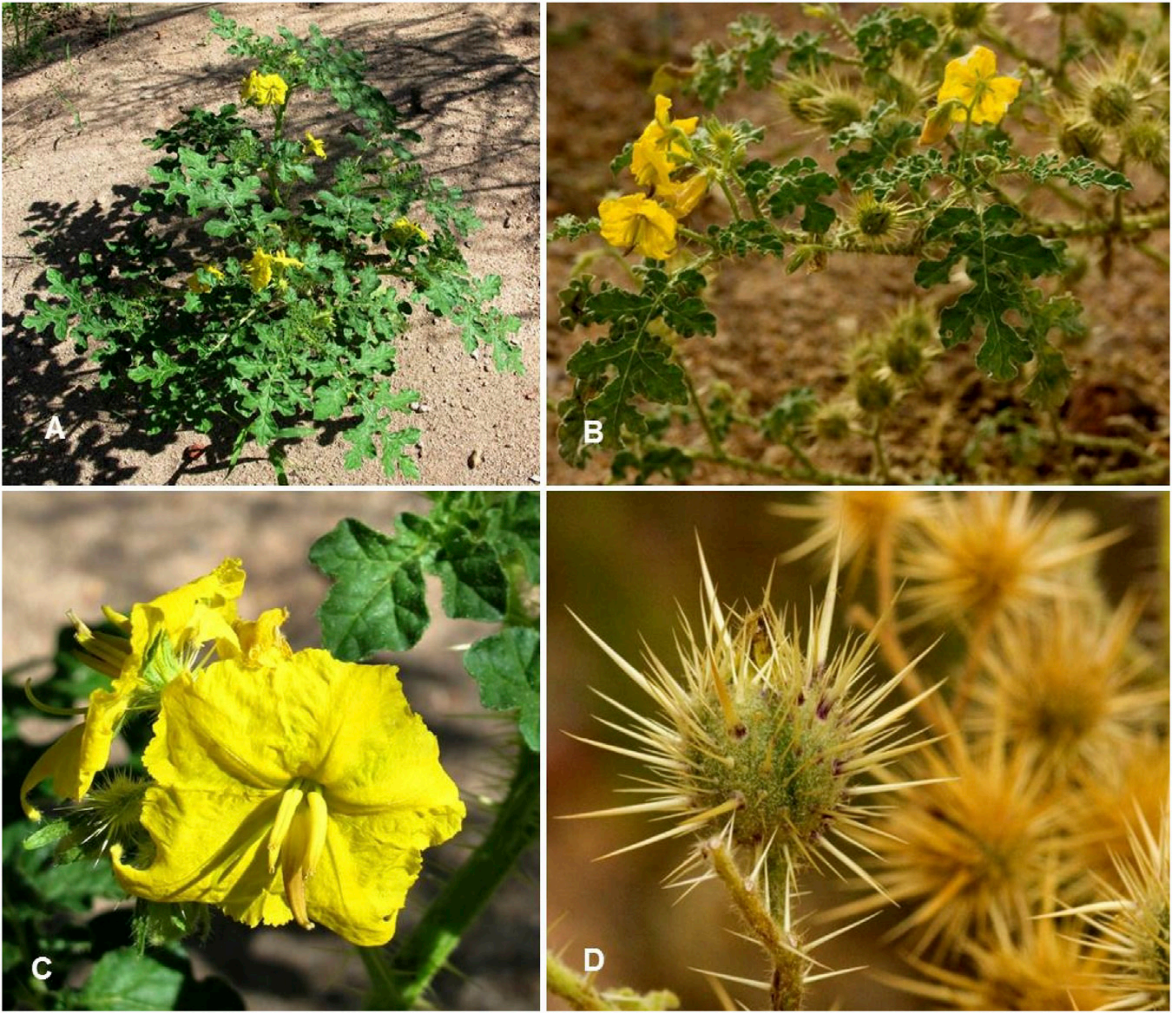
*Solanum rostratum.* **(A)** Habit and leaves [Photo credit by Frankie Coburn (Swbiodiversity, 2023a)]; **(B)** Inflorescence and immature infructescence [Photo credit by Patrick Alexander (Swbiodiversity, 2023b)]; **(C)** Inflorescence [Photo credit by Frankie Coburn (Swbiodiversity, 2023c)]; **(D)** Mature fruits [Photo credit by Patrick Alexander (Swbiodiversity, 2023d)].

*Solanum rostratum* Dunal. Hist. Nat. Solanum: 234 (1813) (Table 1; Figure 7)

Synonyms: *Androcera rostrata* (Dunal) Rydb., *Nycterium rostratum* (Dunal) Link.

Description: Annual herbs that are seated with thorns that resemble needles and pubescent with stellate hairs. Up to 60 cm high, tall, splayed-branched stem. The petioles are 1–2 times shorter than the plate; the leaves have stellate hairs on both sides and are oblong-ovate to ovate in shape. They are pinnately divided into obovate segments, which are then divided into rounded-oblong lobes 7–10 cm long and 4–7 cm wide. Racemes with three to eight flowers each are made up of flowers on short stalks. Campanulate, 1.5–2.5 mm long, stellate-haired, and two-thirds of its length divided into lanceolate lobes, the calyx is maintained in fruit. Yellow, 3–4 mm in diameter, slightly zygomorphic corolla with ovate-lanceolate lobes. Nearly equal in length, the fifth anther is much longer and more strongly bent. Berry drying out and cracking inside a growing cup. Seeds are brown, unevenly angular, and fine-meshed (BSBI Species Accounts Archive, 2010).

Phenology: Flowers in August, fruits in September.

Reproduction: By seeds.

Population status: Common, found in dense groups.

Global distribution: Africa (Libya, Morocco, Tunisia, South Africa). Asia (Azerbaijan, Bangladesh, China, India, Japan, Kazakhstan, Palestine, South Korea, Uzbekistan). Europe (Albania, Austria, Belgium, Bulgaria, Denmark, France, Germany, Hungary, Ireland, Latvia, Lithuania, Moldova, Norway, Russia, Slovakia, Ukraine, United Kingdom). North America (Canada, Mexico, United States). Oceania (Australia, New Zealand).

Habitat: *S. rostratum* occurring in roadsides, disturbed grounds, lowland river valleys and sides of irrigation canals, built-up areas, and agricultural lands.

Invasiveness: *S. rostratum* is a fast-growing, vigorous weed native to North-Central America, South America, and it includes the countries Panama, Costa Rica, Nicaragua, Honduras, El Salvador, Guatemala, and Belize. Now widely introduced or migrated into several countries of Europe, Asia, South Africa, and Australia. The species invade ecosystems by forming dense colonies, and a single plant can produce hundreds of seeds (Vallejo-Marin, 2010) which are dispersed by both biotic and abiotic vectors and self-propelled by its dehiscent fruit. The species is a declared noxious weed in Central Asia and is listed as invasive alien plant species in Uzbekistan.

Phytochemistry and Pharmacology: Various studies have shown that *S. rostratum* contains alkaloids, flavonoids and steroids (Chang et al., 2017; Huang et al., 2017; Omar et al., 2018). Omar et al. (2018) reported the isolation and structure elucidation of linalyl-β-glucopyranoside and apigenin-7-O-glucoside. Liu et al. (2021b) isolated pyrrole alkaloids from the leaves of *S. rostratum* and identified three pairs of novel enantiomeric pyrrole alkaloids: (2′*R*)-caffeicpyrrole A, (2′*S*)-caffeicpyrrole A, (2′*R*)-caffeicpyrrole B, (2′*S*)-caffeicpyrrole B, (2′*R*)-caffeicpyrrole C, and (2′*S*)-caffeicpyrrole C. Authors such as Omar et al. (2018) and Valadez Vega et al. (2019) demonstrated the antioxidant and anti-carcinogenic effects of *S. rostratum*.

**FIGURE 8:**
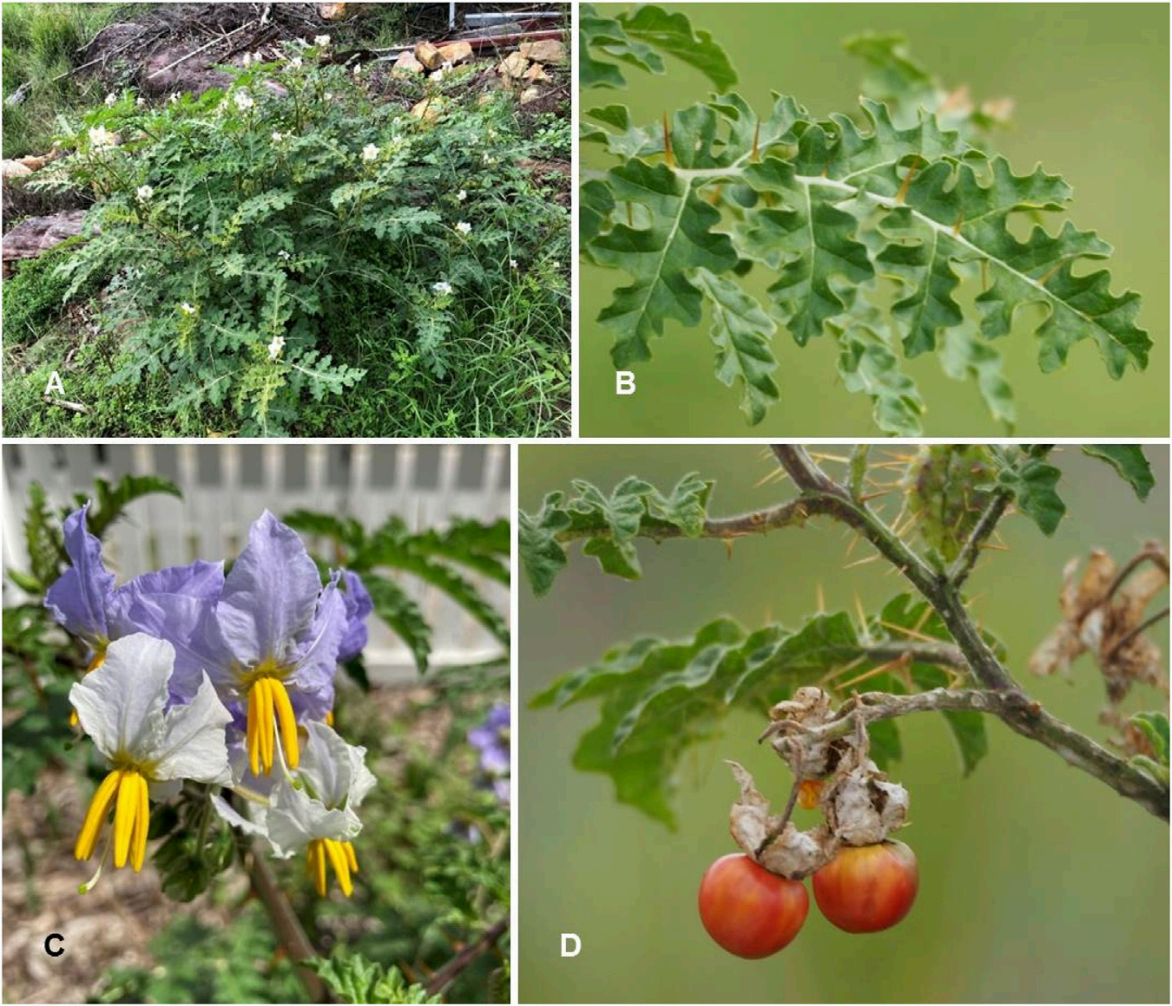
*Solanum sisymbriifolium*. **(A)** Habit [Photo credit by Inaturalist (2023d)]; **(B)** Leaves [Photo credit by Inaturalist (2023c)]; **(C)** Inflorescence [Photo credit by Phillip Mayhair (Inaturalist, 2023a)]; **(D)** Fully mature fruits [Photo credit by Inaturalist (2023b)].

*Solanum sisymbriifolium* Lam. Tabl. Encycl. 2: 25 (1794) (Table 1; Figure 8)

Synonyms: *Solanum balbisii* Dunal, *S. balbisii* var. *bipinnata* Hook, *S. balbisii* var. *oligospermum* Sendtn, *S. balbisii* var. *purpureum* Hook, *S. bipinnatifidum* Larrañaga, *S. brancaefolium* J. Jacq, *S. decurrens* Balb, *S. edule* Vell. nom. illeg., *S. formosum* Weinm., *S. inflatum* Hornem., *S. mauritianum* Willd. ex Roth, *S. opuliflorum* Port. ex Dunal, *S. pilosum* Raf., *S. rogersii* S. Moore, *S. sabeanum* Buckley, *S. sisymbriifolium* f. *albiflorum* Kuntze, *S. sisymbriifolium* var. *bipinnatipartitum* Dunal, *S. sisymbriifolium* var. *brevilobum* Dunal, *S. sisymbriifolium* var. *gracile* Mattos, *S. sisymbriifolium* var. *heracleifolium* Sendtn, *S. sisymbriifolium* f. *lilacinum* Kuntze, *S. sisymbriifolium* var. *macrocarpum* Kuntze, *S. sisymbriifolium* var. *oligospermum* (Sendtn.) Dunal, *S. sisymbriifolium purpureiflorum* Dunal, *S. subviscidum* Schrank, *S. thouinii* C. C. Gmel., *S. viscidum* Schweigg, *S. viscosum* Lag, *S. xanthacanthum* Willd.

Description: Annual herbs with enormous needle-like spines and pubescent stellate with sticky glandular hairs. 50–150 cm tall erect, branching stem. On petioles studded with spines and 2–3 times shorter than the blade, the leaves are elliptical, whole or pinnately divided into oblong serrated segments, 5–10 cm long, and 3–5 cm wide. The leaves also have stellate hairs on both sides. Racemose inflorescences of flowers on long stalks. Calyx campanulate, 1 cm long, roughly half-carved into linear-triangular lobes, preserved throughout fruiting, significantly enlarged and ripped into five turning sections. White or bluish corolla with a limb divided into ovoid lobes, 3–4 mm in diameter, regular, glabrous on the outside with stellate hairs. Five anthers, each equal. The berry is 1–2 cm in diameter and bright crimson inside a growing calyx: Brown, kidney-shaped seeds.

Phenology: Flowers in September, fruits in October.

Reproduction: By seeds.

Population status: By seeds and rhizomes.

Global distribution: Africa (Benin, Kenya, Morocco, South Africa). Asia (China, India, Republic of Korea, Uzbekistan). Europe: (Austria, Belgium, Czech Republic, Denmark, Germany, Ireland, Estonia, Finland, France, Hungary, Italy, Latvia, Lithuania, Netherlands, Norway, Portugal, Spain, Sweden, Turkey, United Kingdom, Ukraine). North America (Canada, United States). South America (Argentina, Bolivia, Brazil, Chile, Colombia, Ecuador, Paraguay, Peru, Uruguay, Venezuela). Oceania and Western Australia.

Habitat: This species occurs in agricultural lands and includes irrigated crops and pastures. The species grows in ruderal and disturbed habitats in urban and semi-urban areas. The species also grows in coastal areas, roadsides, disturbed grounds, lowland river valleys, and built-up areas.

Invasiveness: *Solanum sisymbriifolium* is native to South America and has been introduced into other regions for ornamental purposes. However, as *S. sisymbriifolium* tends to be invasive, its introduction as a trap crop or cultivated plant into a new region should be considered thoroughly before implementation.

Phytochemistry and Pharmacology: *S. sisymbriifolium* is an important source of alkaloids (cuscohygrine, solacaproine, solamine, solasodiene and solasodine), phenolics (caffeic acid, chlorogenic acid, dihydrocaffeic acid, ferulic acid), flavonoids (kaempferol-3-rutinose, rutin), and saponins as isonuatigenin-3-*O*-β-solatriose (Ibarrola et al., 1996; Ferro et al., 2005; Gupta et al., 2014; More, 2019). Figueiredo et al. (2021) identified steroidal saponins as nuatigenin-3-*O*-β-chacotriose (nuatigenoside) from the roots of *S. sisymbriifolium*.

Biological activities of different parts of *S. sisymbriifolium* were reported including anticancer, anti-diabetic, antimicrobial, antioxidant, hepatoprotective, and hypotensive properties (Ibarrola et al., 1996; Ferro et al., 2005; Gupta et al., 2014; More, 2019; Gebrewbet et al., 2023).

**FIGURE 9:**
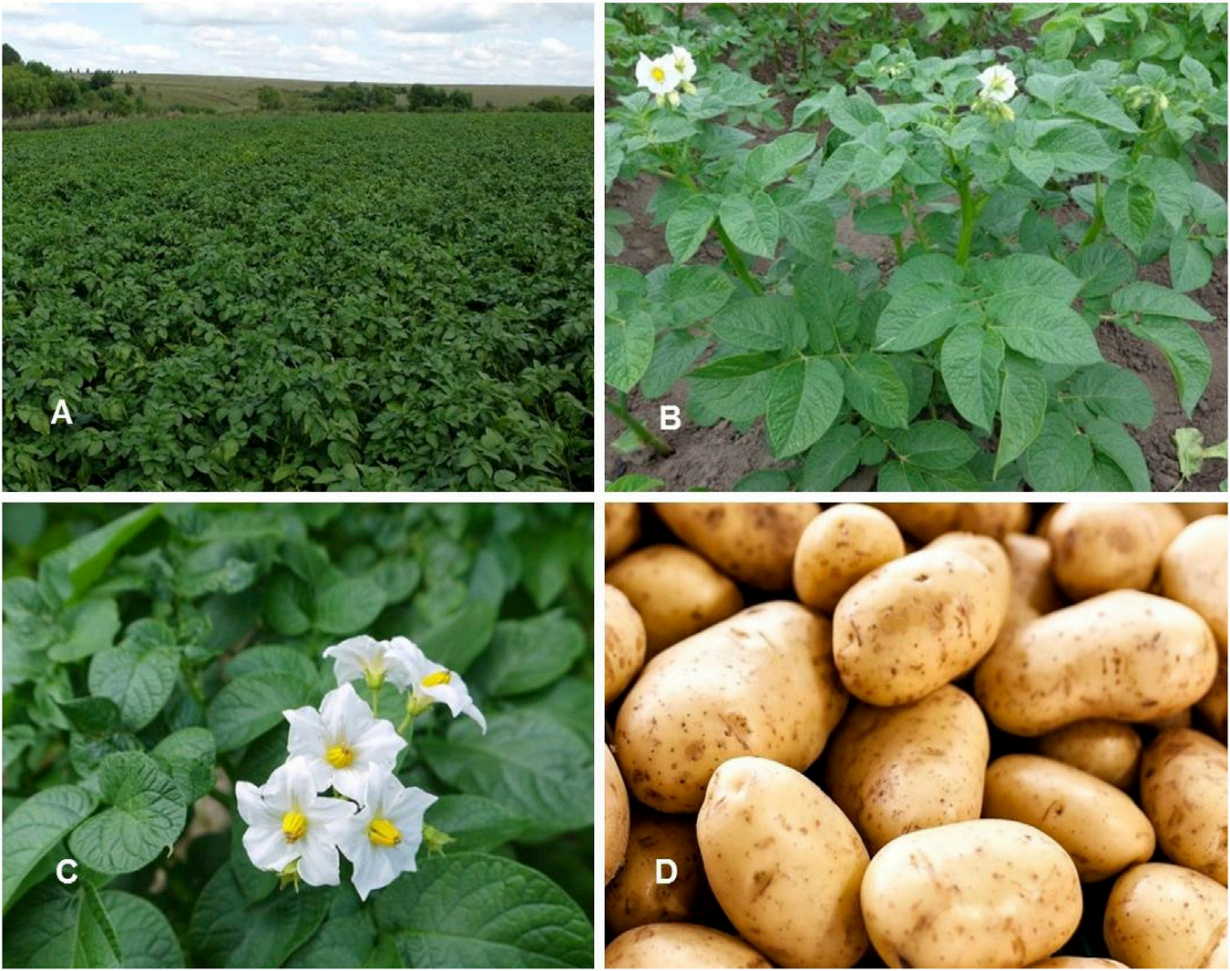
*Solanum tuberosum*. **(A,B)** Habit and leaves; **(C)** Inflorescence; **(D)** Fully mature fruits (Tashkent province, Qibray district, early potato farmer area). Photo credit by Yusufjon Gafforov and Trobjon Makhkamov.

*Solanum tuberosum* L. Sp. Pl.: 185 (1753) (Table 1; Figure 9)

Synonyms: *Lycopersicon tuberosum* (L.) Mill., *Solanum tuberosum* var. *cultum*, nom. superfl., *S. tuberosum* var. *vulgare* Hook. f., nom. inval.

Description: Herbs that are 30–80 cm tall, erect or spreading, glabrous or sparingly pubescent, and have simple and glandular hairs. Stolons harbouring underground tubers that can be white, scarlet, or purplish and are fleshy, globose, oblate, or elliptic in shape. Petiole 2.5–5 cm; leaflet blade oval or oblong, typically sparingly pilose; interrupted odd-pinnate leaves with 6–8 pairs of leaflets and smaller, uneven interstitial leaflets; Panicles that resemble terminal, leaf-opposing, or axillary inflorescences have several flowers and few stems. From mid, the pedicel articulates by 1–2 cm. The lanceolate, sparsely pubescent calyx lobes. Corolla spins measure 2.5–3 cm in diameter and have deltate lobes (5 mm long). It could be white, blue-purple, pink, or purple. The anthers are 5–6 mm in length; the evident ovaries are about 8 mm in style, and the filaments are around 1 mm. Berry green or yellowish green, smooth, globose, frequently striped and about 1.5 cm in diameter (Stern et al., 2013; Solanum tuberosum, 2023).

Phenology: The time of flowering and fruiting depends on early or late varieties of potatoes and it depends on which region of Uzbekistan it is planted. Most potatoes were planted in the Samarkand area on almost 17,000 ha of land in 2017, followed by the Tashkent region (almost 15,000 ha in 2017) (Netherlands Enterprise Agency, 2020). Two harvests per year are carried out in Uzbekistan, namely, first the spring harvest from February to June and the shorter autumn harvest from the end of July to the end of October.

Reproduction: By tuber, rhizomes and seeds.

Population status: Common.

Global distribution: *S. tuberosum* is native to South America (Argentina, Bolivia, Chile, Colombia, Ecuador, Peru, and Venezuela). It is cultivated worldwide in over one hundred countries throughout Africa, Asia, Australia, Europe, and North America.

Habitat: Cultivated *S. tuberosum* cultivars are planted on agricultural lands.

Phytochemistry and Pharmacology: Secondary metabolites in *S. tuberosum* tubers include both phytonutrients and various secondary metabolites. Camire et al. (2009) reported that *S. tuberosum* is an important source of vitamins (vitamin C, B_1_, B_2_, B_3_, B_5_, B_6_, E, folic acid, β-carotene, etc.), and minerals as Fe, Mg, P, and Zn. Furthermore, Drewnowski and Rehm (2013) reported that *S. tuberosum* is the most affordable source of vitamin C, potassium and fibre providing about 10% of its daily value. Anjum Sahair et al. (2018) reported phenolic derivatives as chlorogenic acid, caffeic acid, gallic acid and protocatechuic acid. The protein content of *S. tuberosum* is comparable to that of cereals, and nutritionally, potato protein is similar to that of whole eggs (Franková et al., 2022). Among the secondary metabolites, glycoalkaloids are considered as the most common. Based on the literature data (Shakya and Navarre, 2008), various cultivars of *S. tuberosum* contain less than 20 mg/100 g f.w. of total glycoalkaloids, whereas chaconine and solanine are thought to comprise up to 90% of the total glycoalkaloid content of domesticated ones, with chaconine frequently being more prevalent than solanine. According to Fogelman et al. (2019), the tuber of *S. tuberosum* contains various secondary metabolites, including anthocyanidins such as peonidin and pelargonidin, carotenoids primarily composed of xanthophylls, phenolic acids: caffeic acid and coumaric acid, along with a few unidentified acids structurally similar to chlorogenic, hydroxycinnamic, and coumaric acid. Additionally, the tuber harbors flavonol, specifically kaempferol, as well as toxic steroidal glycoalkaloids (SGAs). Two years afterward, in 2021, Sampaio et al. (2021) conducted an analysis of ten varieties of colored potato peels, revealing the presence of both non-anthocyanin and anthocyanin phenolic compounds in the examined samples. Among the non-anthocyanin phenolics, caffeic and caffeoylquinic acid were present in the highest concentrations across all samples. Additionally, *O*-glycosylated flavonoid derivatives and polyamine derivatives were detected. In terms of anthocyanins, all tentatively identified compounds were acylated with a hydroxycinnamic acid. In the same study, the researchers observed that all examined samples exhibited both antioxidant and antitumor activities, demonstrating no adverse effects. The *Rosemary* variety extract of *S. tubersoum* displayed the most favorable results in terms of antioxidant and antitumor effects and was the sole sample to exhibit anti-inflammatory activity (Sampaio et al., 2021).

Recently, Baur et al. (2022) showed the light impact on quantitation of toxic steroidal glycoalkaloids and identification of newly identified saponins from *S. tuberosum* tubers.

The wide variety of phytonutrients found in potatoes, including anthocyanins, carotenoids, minerals, polyphenols and vitamins, have the potential to improve human health and diet (Mishra et al., 2020; Franková et al., 2022; Kowalczewski et al., 2022). Nutritional value of *S. tuberosum* was highlighted by authors (Fogelman et al., 2019; Dereje et al., 2021). Being rich in carbohydrates, vitamins and antioxidants, the potato is a staple food and potato starch has unique properties compared to cereal starches (Xu et al., 2023). In addition, Rosas-Cruz et al. (2020) postulated that the wound healing mechanism of *S. tuberosum*-based ointment is related to phytoconstituents as phenolic compounds that exert antioxidant, antimicrobial, and anti-inflammatory effects, which contributes to the optimal healing process.

**FIGURE 10:**
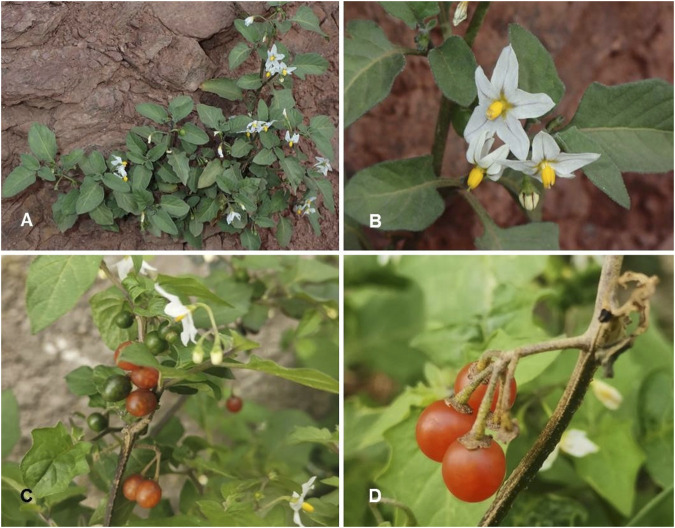
*Solanum villosum.* **(A)** Habit and leaves (Photo credit by Julien Renoult); **(B)** Inflorescence [Photo credit by Julien Renoult (Inaturalist, 2023f)]; **(C)** Inflorescence and immature infructescence (Photo credit by Corentin Desseux); **(D)** Fully mature fruits [Photo credit by Corentin Desseux (Inaturalist, 2023e)].

*Solanum villosum* Mill. Gard. Dict. ed. 8.: n. 2 (1768) (Table 1; Figure 10)

Synonyms: *Solanum luteum* subsp. *villosum* (Mill.) Dostál

Description: Grayish annual herbs having stem 15–70 cm high, erect or, less frequently, decumbent, splayed-branched from the base up, cylindrical in the bottom half, higher like the branches, not usually tetrahedral, indistinctly ribbed and especially densely covered in short, upward-facing hairs when young. The petiole’s blades are shorter than the leaves because the leaves are bluish-greenin young stage, densely pubescent on both sides with short, semi-appressed hairs, later becoming bare, ovate, oblong-ovate or rhombic-ovate, obtuse at the apex or gradually narrowed from the middle, with almost rounded or more frequently wedge-shaped leaves, descending to the petioles. Corymbose, extra-axillary, and 4–8 flowered inflorescences. Campanulate, subappressedly pubescent, ranging in length from 2 to 2.5 mm, with a third bluntly toothed. Corolla is white, 4.5–5 mm in diameter triangular ovate limb lobes, and is externally somewhat pubescent. Berry is spherical, orange-red or brownish-red, and 7–10 mm in diameter. Berry is spherical, orange-red or brownish-red, and 7–10 mm in diameter. Yellow, reniform, 2 mm long, and fine-meshed seeds are produced (Solanaceaesource, 2023).

Phenology: Flowers in June - October, fruits in July–September.

Reproduction: By seeds.

Population status: Common, found in small groups.

Global distribution: Africa (Algeria, Angola, Burundi, Egypt, Eritrea, Ethiopia, Kenya, Libya, Madeira, Malawi, Morocco, Mozambique, Nigeria, Somalia, Sudan, Tanzania, Tunisia, Uganda), Asia (Afghanistan, Bangladesh, China, India, Iran, Iraq, Italy, Kazakhstan, Kirgizstan, Korea, Myanmar, Nepal, Oman, Pakistan, Palestine, Saudi Arabia, Tajikistan, Taiwan, Turkmenistan, Uzbekistan, Vietnam, Yemen, Zambia, Zimbabwe), Europe (Albania, Austria, Belgium, Belarus, Bulgaria, Denmark, France, Germany, Greece, Ireland, Hungary, Netherlands, Poland, Portugal, Romania, Serbia, Slovakia, Slovenia, Spain, Sweden, Switzerland, Turkey, United Kingdom, Ukraine), North America (United States), Oceania (Australia, New Zealand).

Habitat: *Solanum villosum* is a prevalent species found on roadsides, disturbed grounds, mountain river valleys and lakesides, lowland river valleys and sides of irrigation canals, built-up areas, and agricultural lands.

Food: *S. villosum* is more commonly cultivated in eastern Africa, and many specimen labels note that the fruits of *S. villosum* are particularly prized by children (Keding et al., 2007).

Phytochemistry and Pharmacology: Numerous phytochemicals as alkaloids, amino acids, carbohydrates, fatty acids, flavonoids, glycosides, phenols, proteins, saponins, steroids, tannins, and terpenoids were identified from *S. villosum* (Venkatesh et al., 2014a; Chowdhury et al., 2015; Ben-Abdullah et al., 2018; Zahara et al., 2019). Ahmad et al. (2019) summarized that leaves of *S. villosum* contain high levels of nutrients such as carbohydrates and proteins, minerals (Ca, Fe, and P), and vitamins (especially vitamins A, B and C). According to Wojdyło et al. (2019), recommended daily intake for Ca is 1,200 mg for adults, and Ca content in *S. villosum* is 442 mg/100 g d.w. which was higher than reported in other *Solanum* species, i.e., *S. retroflexum* (199 mg/100 g d.w.). Furthermore, Ben-Abdullah et al. (2018) reported for the first time the impact of salt stress induced by NaCl on the production of carotenoids (β-carotene, lutein), glycoalkaloids (GAs) (β-solamagine, α-solasonine), and phenolic compounds (caffeic acid, quercetin, quercetin 3-β-D-glcoside) in *S. villosum*.

Based on traditional medicine in Southern India, free radical scavenging activity was reported for *S. villosum* (Venkatesh et al., 2014b). Furthermore, according to Zahara et al. (2019), *S. villosum* has high nutritional value and used as an Ayurvedic herb with multiple medicinal properties (antifibrotic, antimicrobial and hepatoprotective activity), it is a good source of pharmaceutical agents such as steroidal alkaloids, phenolic compounds, saponins, etc. Indeed, the hydromethanol immature fruit extract possess a high molluscicidal activity against *Galba truncatula* intermediate host of trematode *Fasciola hepatica*, a causal agent of fascioliasis in humans (Chowdhury et al., 2008). Chloroform and methanol extracts of *S. villosum* green berries showed a potential larvicidal biocontrol activity against *Aedes aegypti* (= *Stegomyia aegypti*), mosquito that can mainly spread dengue fever, chikungunya, Zika fever and yellow fever (Chowdhury et al., 2008). Two years later, Abdel-Sattar et al. (2010) reported that methanolic, petroleum ether, chloroform, ethyl acetate and aqueous extracts of *S. villosum* showed significant antiprotozoal activity against *Plasmodium falciparum*, *Trypanosoma brucei*, *T. cruzi* and *Leishmania infantum*. On the other hand, glycoalkaloids (solanine and solasodine) from butanol extract of *S. villosum* fruit possessed anticancer potential on LIM-1863 human colon carcinoma cell line (Venkatesh et al., 2014a). Five years later, Nyaga et al. (2019) reported the antidiabetic property of this plant in a streptozotocin-induced diabetic mice model. The authors proposed that the presence of flavonoids, alkaloids, tannins, saponins, phenols, and glycosides in the plant makes it a potential candidate for novel diabetes therapies, especially considering its demonstrated lack of toxicity. A crude extract comparison showed *in vitro* antimicrobial activities of *Solanum villosum* (AbdelGawwad et al., 2020). Recently, Staveckiene et al. (2023) demonstrated the effect of ripening stages on the accumulation of polyphenols and antioxidant activity of the fruit extracts of *S. villosum*.

### Ethnobotanical study and uses of the eight *Solanum* species from Uzbekistan

In the 21st century’s third decade, scientists are still making remarkable strides in the domain of ethnopharmacology and research related to different kinds of medicinal plants (Kim et al., 2023). Regarding this, the World Health Organization reported that indigenous or native populations worldwide still practice traditional medicine using plants as their primary source of treatment and have built their medicinal systems based on their theories, beliefs, and experiences (Mir et al., 2021; Nanjala et al., 2022). Before the modern scientific practice, the traditional herbal medicine was used in healthcare, and most people worldwide still rely on herbal health practices today (Das and Barua, 2013). Recently, more than 80% of the people in Asian and African countries depend on it for primary healthcare (Nanjala et al., 2022). However, despite herbs being used as effective medicines for centuries, the majority lack scientific support and are unexplored. A major approach in ethnopharmacology—ethnobotany, ethnomycology and ethnozoology in particular—is how to transmit knowledge in these fields over a long period, over a large geographical area, or between geographically, culturally or scientifically different regions of the world (Cunningham and Yang, 2011; Fuller, 2013; Guerrero-Gatica et al., 2020). Therefore, ethnobotanists can help rescue this disappearing knowledge and return it to local communities (Ramachandran et al., 2009; Ford, 2015; Belichenko et al., 2022). In general, ethnobotany is the study of the direct interrelationship between human beings and plants, which examines how human communities have used plants to meet their spiritual and practical requirements (Galvis-Tarazona et al., 2022). To ensure the sustainability of plants in the future, it is also crucial to understand the socio-ecological dynamics surrounding their current distribution, trade, and protection (Kunwar et al., 2020).

*Solanum* is a globally distributed genus; it is rich in various classes of bioactive metabolites and has been used by different tribes all over the world for centuries in traditional medicine and human nutrition (Anjum Sahair et al., 2018; Chidambaram et al., 2022). Central Asia includes five countries: Kazakhstan, Kyrgyzstan, Tajikistan, Turkmenistan, and Uzbekistan, with about 9,800 vascular plant species and among them, Uzbekistan with over 4,500 species, has a central position in the region (Khojimatov et al., 2020). About 600 plant species have been used in traditional medicine, but only about 200 species have been phytochemically characterized, and some of them (about 150 species) were included in the original Pharmacopoeia of Uzbekistan (Khojimatov et al., 2020).

Although traditional medicine in Central Asia has a vast history that dates back many centuries, its most notable era was between the 10th and 11th centuries (Tayjanov et al., 2021; Gafforov et al., 2023). Abū-ʿAlī al-Ḥusayn Ibn-ʿAbdallāh Ibn-Sīnā (Avicenna, 1930) (arab. ابن سینا, Ibn Sina) was born in 980 in the village of Afshana, present day Bukhara region in Uzbekistan and died in 1037 in Hamadan (Tayjanov et al., 2021). He was one of the early scientists exploring folk medicines’ secrets in medieval East. The “Book of Healing” (Kitab al-Shifa), followed by “The Canon of Medicine” were both written by Avicenna. “The Canon of Medicine” (arab. القانون في الطب, Al-Qānūn fī al-ṭibb) the main medical work of Avicenna is a genuine medical encyclopedia. For many centuries, the work of Avicenna served as the main medical guide of many countries. The Canon was divided into five books. First book of Canon covered general medical and physiological principles and general therapeutic procedures as well. The second book of the Canon is an encyclopedia of medicines (Materia Medica). Descriptions of about 800 therapeutic compounds derived from plants, minerals, and animals may be found. These descriptions were developed on the basis of the identification and management of diseases that affect the entire body. Disorders of various organs were covered in the chapter of the third book on specific pathology. Fifth book is kind of pharmacopoeia and contains lists of about 650 medicinal compounds including their uses and medicinal effects. In general, it combines the experiments of medicine of ancient. Of the 810 drugs listed in the book of Canon, 515 are medicinal plants (and their agents), 125 products of animal origin, and 85 minerals (Tayjanov et al., 2021). Also, of the selected *Solanum* species, Avicenna documented the traditional use of *S. nigrum* in the form of juice as the eye remedy in the conditions where children’s eyes swelled after crying, and hepatoprotective agent (Table 2).

**TABLE 2:** Ethnobotanical, ethnopharmacological and food uses of the eight *Solanum* species from Uzbekistan (species name, plant part, country, uses, mode of administration, and used references).

Species	Plant part used	Continent/Country	Uses	Preparation and mode of administration	References
*Solanum dulcamara*	Shoots	—	Depurative properties, treat eczema, gout, herpes, pityriasis, and rheumatism	Boil 20–30 g of dried shoots in 1 L water and drink over 3 days between meals	TF03 Solanum dulcamara L (2023)
*S. dulcamara*	Leaves	—	Relieve sprains and help with hemorrhoids	Leaves boiled in wine	TF03 Solanum dulcamara L (2023)
*S. dulcamara*	Leaves	—	Remove facial blemishes	Fresh juice of leaves	TF03 Solanum dulcamara L (2023)
*S. dulcamara*	Leaves	Ethiopia	Used for treating wounds	Crushing fresh leaves, whereas wrapping takes 2 days	Asfaw et al. (2023)
*Solanum lycopersicum*	Leaves	Zimbabwe	Animal eye problems	Animals made to drink a mixture of crushed leaves and water	Maroyi (2012)
*S. lycopersicum*	Unripe fruits	Albania and North Macedonia	Food	Lacto-fermented in water and salt	Pieroni et al. (2014)
*S. lycopersicum*	Unripe Fruits	Uzbekistan	Used for treating varicose veins	The reason why blue *S. lycopersicum* was chosen is that it contains solanine, which vanishes when the fruit is fully mature. The varicose vein was tied with a raw tomato that had been cut into two equal halves. After two to 3 minutes, it was taken out	Gynecology (2023)
*S. lycopersicum*	Fruits	Uzbekistan	Used to reduce the risk of developing oncological diseases of the prostate, mammary glands, pancreas and ovaries	Fresh juice	Gepamed (2023)
*S. lycopersicum*	Fruits	Uzbekistan	Used to lower blood pressure	Fresh juice	Gepamed (2023)
*S. lycopersicum*	Fruits	Uzbekistan	It was very helpful for people with erectile dysfunction. Consuming enough tomatoes can improve impotence, increase sperm count, and sperm motility	Fresh fruits, and cooked fruits in olive oil	Gepamed (2023)
*S. lycopersicum*	Fruits	Uzbekistan	Used to prevent heart disease and stroke	Fresh juice	Gepamed (2023)
*S. lycopersicum*	Fruits	South and Central America	Used as antibiotic, anti-inflammatory and diuretic agent	—	Buabeid et al. (2022)
*S. lycopersicum*	Fruits and leaves	Uzbekistan	Tomato juice is used to treat ulcers, purulent wounds, liver diseases and prevention of vitamin deficiencies	Fresh juice, and decoction of leaves, dried or fresh tomato tops. Freshly squeezed tomato juice can be used together with honey in treatment of liver diseases	Solanum-Lycopersicum (2023)
*Solanum melongena*	Leaves	India	Used against fever	Fresh juice	Mutalik et al. (2003)
*S. melongena*	Various parts of the plant	India	Used against asthma, bronchitis, cardiac debility, cholera, inflammatory conditions, neuralgias, and ulcer of nose	—	Mutalik et al. (2003), Das and Barua (2013)
*S. melongena*	Fruits	Trinidad and Tobago	Urinary problems caused by high cholesterol levels (validation score not proven)	—	Lans (2006)
*S. melongena*	Peduncle	Lebanon	Used for gum inflammation	—	Diab et al. (2011)
*S. melongena*	Fruits	Albania	Food	Cooked	Pieroni et al. (2014)
*S. melongena*	Roots	China	Used in the cases of beriberi, blood in the stool, chilblains, pruritus, toothache, and wind-damp-heat syndrome	—	Sun et al. (2014), Yang et al. (2019), Liu et al. (2021a)
*S. melongena*	Fruits	Morocco	Used as anti-hypercholesterolemic plant	Infusion	Arrout et al. (2022)
*S. melongena*	Fruits	Uzbekistan	It is applied in cases of atherosclerosis, cardiovascular diseases, edema, as well as conditions related to the liver, kidneys, and gastrointestinal system	Infusion and juice of fresh fruits	Solanum-Melongena (2023)
*Solanum nigrum*	Leaves and fruits	Uzbekistan	Used as the eye remedy with cooling effect in the cases when children’s eyes swell after crying and in the cases when a thorn appears on their pupils and hepatoprotective agent. Hepatoprotective application of *S. nigrum* is based on the treatment which combined application of nightshade juice, bubble cherry juice, celery juice, ragwort juice and dandelion juice	Squeezed juice and nightshade juice prepared in combination with some other plants	Avicenna (980–1037), 1930
*S. nigrum*	Fruits	Algeria	Used in the cases of blindness, cataract, conjunctivitis, glaucoma, and trachoma	Diluted infusion of berries	Boulos (1983)
*S. nigrum*	Whole plant	Algeria	Used in the cases of burns and dermal affections	Decoction	Boulos (1983)
*S. nigrum*	Leaves	India and Algeria	Remedy in the cases of blood coagulation, diabetes, heart problems, indigestion, jaundice and skin diseases	—	Boulos (1983), Padalia (2014)
*S. nigrum*	Fruits	Algeria	Blood coagulation, heart diseases, jaundice, liver diseases and stomachache	—	Boulos (1983)
*S. nigrum*	Fruits	North Africa	Treatment of blindless, conjunctivitis, glaucoma, trachoma and cataract	Diluted infusion	Boulos (1983)
*S. nigrum*	Leaves	Israel	External wounds	1) Ash of burnt leaves applied on the wound; 2) Cataplasm of crushed fresh leaves; 3) Cooked leaves are applied; 4) Extracted leaf juice is applied	Dafni and Yaniv (1994)
*S. nigrum*	Leaves	Israel	Hemorrhoids	External cataplasm of crushed leaves is applied	Dafni and Yaniv (1994)
*S. nigrum*	Leaves	Israel	Heart and liver diseases	Infusion as a drunk	Dafni and Yaniv (1994)
*S. nigrum*	Leaves	Israel	Used as a sedative for external pains and aches (backaches, chestaches) and against Scabies	Crushed leaves are massaged on the affected organ	Dafni and Yaniv (1994)
*S. nigrum*	Leaves	Israel	Burns	Cataplasm of crushed leaves in olive oil	Dafni and Yaniv (1994)
*S. nigrum*	Fruits	Israel	Toothache	Fruits are boiled with buttermilk and applied on teeth, or cooked fruits are applied	Dafni and Yaniv (1994)
*S. nigrum*	Whole plant	Congo	Snake bite or sting by venomous animals	Maceration of the whole plant and oral administration	Chifundera (1998)
*S. nigrum*	Fruits	Mexico	Remedy for the treatment of nervous conditions	A tonic was prepared of the fruit, which was boiled in water to yield a clear yellowish liquid, then a small cup was drunk daily	Pérez et al. (1988)
*S. nigrum*	Aerial parts	Italy	Analgesic, antispasmodic, and sedative remedy. Sliced fresh pulp externally applied for skin diseases, itching and painful joints	—	Leporatti and Ivancheva (2003)
*S. nigrum*	Leaves	India	Used as a liver tonic and in cases of indigestion	Tonic	Kala (2005)
*S. nigrum*	Root	Nepal	Used in intermittent fever and easy child delivery	Amulet of roots	Acharya and Pokhrel (2006)
*S. nigrum*	Plant	Malaysia	Used to treat fever, inflammation, and pain	—	Zakaria et al. (2006)
*S. nigrum*	Fruits	Yemen	Used as skin antiseptic, expectorant, laxative, and for treatment of diarrhea and hemorrhages	—	Al-Fatimi et al. (2007)
*S. nigrum*	Root	India	Increase fertility in women	The roots with a small amount of sugar are boiled in water	Parveen et al. (2007)
*S. nigrum*	Root	India	Treatment of asthma and whooping cough	Extract pure juice	Sikdar and Dutta (2008)
*S. nigrum*	Root	India	Used against asthma and whooping cough	The juice of the roots is extracted	Sikdar and Dutta (2008)
*S. nigrum*	Fruits	Jordan	Antispasmodic and anti-rheumatic drug	—	Al-Qura’n (2009)
*S. nigrum*	Whole plant	Tunisia	Treatment of erysipelas, acute bacterial infection induced by *Staphylococcus* sp. and *Streptococcus* sp., and for eczema	Sap	Leporatti and Ghedira (2009)
*S. nigrum*	Leaves	Tanzania	Topically applied in the treatment of ringworm and dressing of warts as well	Leaves are pounded and applied topically, or in the second case, pounded and baked	Moshi et al. (2009), Jain et al. (2011)
*S. nigrum*	Fruits	Tanzania	Used for kids to stop bed-wetting	Ripe fruits in edible form	Moshi et al. (2009), Jain et al. (2011)
*S. nigrum*	Tuber	India	Traditionally used as a food	Cooked and eaten along with boiled rice	Ramachandran et al. (2009)
*S. nigrum*	Leaves and whole plant	India	Used in the cases of rabies, stomachache, and stomach ulcer, and for the treatment of wound healing	Fresh leaves cooked with onion bulbs, cumin seeds, or leaf juice can also be taken orally. The whole plant was taken as food	Sivaperumal et al. (2010), Jain et al. (2011)
*S. nigrum*	Fruits	Libya	Used as antispasmodic, diuretic, anti-emetic. Treatment of diarrhea, fever, and eye problems as well as for bleeding	—	Aburjai et al. (2014)
*S. nigrum*	Leaves and stem	India	Highly effective in the various body pain (bone fracture, joint pain, ligament rupture and muscles pain as well) and rheumatism	“Gewai saag” is prepared as an ointment from fresh leaves, soft, young stems, and branches. The ointment could be applied warm, freshly prepared twice a day	Padalia (2014)
*S. nigrum*	Whole plant	Myanmar	Anti-flatulent, and antipyretic remedy, digestion promoter, and against heart and lung diseases	Fresh juice	Aung et al. (2016)
*S. nigrum*	Whole plant	India	Against fever and alleviating pain	Fresh juice	Mukhopadhyay et al. (2018)
*S. nigrum*	Leaves	India	Treatment of skin conditions and rheumatoid and gouty arthritis, and used as anti-tuberculosis. Poultices are reported to cause diaphoresis. Additionally, leaves are utilized for neurological problems, nausea, and dropsy	Used usually as a poultice	Mukhopadhyay et al. (2018)
*S. nigrum*	Fruits and flowers	India	Remedy for cough and erysipelas (specific, acute, cutaneous inflammatory disease caused by a hemolytic *Streptococcus*)	Juice prepared as a decoction of fresh fruits and flowers	Mukhopadhyay et al. (2018)
*S. nigrum*	Berries	India	Cathartic, diuretic and tonic properties	—	Mukhopadhyay et al. (2018)
*S. nigrum*	Roots	India	Traditional remedy in cases of hepatitis, osteopathy, ophthalmopathy, and rhinopathy	—	Mukhopadhyay et al. (2018)
*S. nigrum*	Leaves	China, India, Southeast Asia and Europe	As a vegetable, but it was thought to be poisonous by association with the deadly nightshade, *Atropa belladonna*	1) Leaves are often cooked in milk to make them less bitter; 2) Leaves are commonly eaten, particularly in southern China	Särkinen et al. (2018)
*S. nigrum*	Fruits	India	Used for toothache	Fruits	Sharma and Dogra (2018)
*S. nigrum*	Aerial parts, leaves and fruits	Iran	Analgesic and sedative, antidepressant, antiparasitic, hypolipidemic, treatment of addiction, anemia, cancer, constipation, diabetes, hemorrhoids, inflammation and edema, and skin diseases, amongst others	Balm, aqueous and methanolic extracts and oral decoction. In addition, the aqueous extract of leaves with its cool and dry temperament, has astringent and restraint effect, so it has been used as a swelling reliever with *Malva sylvestris* L. or some other ingredients	Eskandari et al. (2019)
*S. nigrum*	Leaves and berries	India	Used for the treatment of rheumatic and gouty joints and skin diseases. Used for the treatment of tuberculosis, nausea and nervous disorders	Leaves and berries are habitually consumed as food after cooking with tamarind, onion, and cumin seeds	Sonkamble et al. (2019)
*S. nigrum*	Leaves	India	Wound healing	Extracts	Sonkamble et al. (2019)
*S. nigrum*	Fruits	India	Laxative, and for treating asthma. Used as an appetite stimulant and “excessive thirst.”	Tonic	Sonkamble et al. (2019)
*S. nigrum*	Whole plant	India	Remedy in the cases of fever and reduces pain as well. On ulcers and other skin diseases	Fresh juice	Sonkamble et al. (2019)
*S. nigrum*	Leaves	Libya	Internal treatment as anesthetic, cholagogue, sedative, and for treating convulsions, dysentery, and insomnia. External treatment of wounds and itching	—	Chen et al. (2022)
*S. nigrum*	Fruits	India	Treatment of cough, diarrhea, inflammations and skin diseases	Decoction of berries	Zanit et al. (2022)
*S. nigrum*	Leaves	India	Remedy of rheumatic joints and skin disorders	—	Zanit et al. (2022)
*S. nigrum*	Leaves	Ethiopia	Remedy for wounds	Crushing fresh leaves	Asfaw et al. (2023)
*Solanum rostratum*	Roots	New Mexico	Remedy for a sick stomach, not an emetic	Infusion of the powdered root	Coxe Stevenson. (1915)
*S. rostratum*	Roots	Texas	Used as a stomach ache remedy	The small amount of roots are grounded and mixed with water and used as a drink	Burlage (1968)
*S. rostratum*	Flowers	Mexico	Remedy for coughs	Decoction of flowers	Martinez (1969)
*S. rostratum*	Aerial parts	Mexico	Used as an anti-diarrheic and anti-hypertensive	Aerial parts decoction taken orally	Martinez (1991)
*S. rostratum*	Leaves	Mexico	Used to treat digestive, and kidney disorders, and stomachache	Infusion of leaves was used as a purgative	Valadez Vega et al. (2019)
*S. rostratum*	Leaves	Mexico	Remedy for chronic coughs	Infusion of leaves	Valadez Vega et al. (2019)
*S. rostratum*	Flowers	Mexico	Used against cough and stomach pain	Tea prepared with flowers	Valadez Vega et al. (2019)
*S. rostratum*	Aerial parts	Mexico	Used as an auxiliary in the treatment of uterine cancer and for vaginal washes to control vaginal fluids, disinfect genitals	Infusions	Valadez Vega et al. (2019)
*S. rostratum*	The branch of the plant	Mexico	Used as anti-rheumatic	The branch is water-cooled and applied in baths	Valadez Vega et al. (2019)
*Solanum sisymbriifolium*	Roots	Paraguay	Traditional remedy for asthma, diarrhea, hypertension, inflammation, liver diseases and respiratory and urinary tract infections as well	—	González Torres (1980), Perez and Anesini (1994), Patel et al. (2013)
*S. sisymbriifolium*	Fruits and roots	Argentina and Paraguay	Analgesic, antisyphilitic, contraceptive, diuretic, in the cases of hypertension diseases, hepatoprotective remedy, and food as well	Boil the fruits or eat the raw fruits	Filipoy (1994), Ferro et al. (2005), More (2019)
*S. sisymbriifolium*	Aerial parts	Argentina	Traditional remedy in cases of diarrhea, respiratory and urinary tract infections	—	Perez and Anesini (1994), Ferro et al. (2005), Uddin et al. (2008)
*S. sisymbriifolium*	Flowers	India	Analgesic	—	Ferro et al. (2005)
*S. sisymbriifolium*	Flowers, fruits, roots and leaves	Brazil	Analgesic, antisyphilitic, contraceptive, diuretic, febrifuge, and hepato-protective remedy	—	Ferro et al. (2005), Uddin et al. (2008), More (2019)
*S. sisymbriifolium*	Fruits	Argentina and Paraguay	Food source for the Chorote Indians from north-west Argentina and south-west Paraguay	Boil the fruits or eat the raw fruits as a source of food	Arenas and Scarpa (2007)
*S. sisymbriifolium*	Fragrant fruits	South America	Traditional remedies based on their characteristic fragrance	—	Pasdaran et al. (2017)
*Solanum tuberosum*	Tuber	Croatia	Topical application as anti-headache agent	Sliced and prepared with rye flour and water	Pieroni et al. (2003)
*S. tuberosum*	Tuber	North Macedonia	Externally applied (in slices) for treating eye inflammations or headaches	Fresh	Pieroni et al. (2014)
*S. tuberosum*	Tuber	Europe and South America	Used to treat and heal burns, constipation, cough, hemorrhoids, scurvy, tumors, and warts, to prevent wrinkles on the face, pain, acidity, and swollen gums	Raw juice and skins	Camire et al. (2009)
*S. tuberosum*	Leaves and tubers	Morocco	They help in the cases of different kinds of burn treatment	Heated leaves or tubers	Khabbach et al. (2012)
*S. tuberosum*	Tuber	Albania	Slices of a fresh tuber are externally applied on the forehead to treat headaches. Traditionally consumed boiled with Albanian home-made seasoning mixture named *piprik e shtupun*, fried, or roasted	Fresh, fried, or roasted	Pieroni et al. (2013)
*S. tuberosum*	Young leaves	Albania	Boiled and consumed as vegetables with buttermilk or as filling for pies especially in the past - however one elderly couple confirmed that they also consume them nowadays)	Fresh or boiled	Pieroni et al. (2013)
*S. tuberosum*	Tuber	Albania and North Macedonia	Food	Cooked	Pieroni et al. (2014)
*S. tuberosum*	Flowers	Uzbekistan	Used to relieve inflammation	—	Birmiss (2023)
*S. tuberosum*	Tuber	Uzbekistan	It was used as an excellent remedy for people with stomach problems and frequent constipation. In addition, this product has been used to heal ulcers and prevent the appearance of new wounds	Fresh juice	Birmiss (2023)
*S. tuberosum*	Tuber	Bangladesh	Traditionally used by local people at Santahar Pouroshova of Bogra district in cases of digestive issues, peptic ulcers, rheumatic joint pain, skin rashes and swellings	—	Mahbubur Rahman et al. (2015)
*S. tuberosum*	Tuber	Uzbekistan	It was used for treating headaches due to its hypotensive effects, chronic diseases of the gastrointestinal tract, peptic ulcers of stomach and duodenum and antidiabetic agents. Additionally, potato peels have been utilized in instances of allergies, hypertension, painful shock, and tachycardia	Fresh juice and potato peels	Solanum-Tuberosum (2023)
*Solanum villosum*	Tuber	India	Traditionally used as a food	Cooked and eaten along with boiled rice	Ramachandran et al. (2009)
*S. villosum*	Leaves	Europe, Africa and the Middle East	Remedy in the treatment of eye conditions and swellings	Leaves consumed as spinach (usually boiled, often in milk) and as a pot-herb	Särkinen et al. (2018)
*S. villosum*	Fruits and leaves	India, Kenya and Pakistan	Traditionally used in cases of leucorrhoea, nappy rash, wounds, and a cold sore, as well as an ointment for sore abscess. Also used as a food	—	Zahara et al. (2019)
*S. villosum*	Unripe fruits	Kenya	Treatment of soothe toothache and additionally squeezed on babies’ gums to ease teething ache	Fresh	Zahara et al. (2019)
*S. villosum*	Leaves	Ethiopia	Remedy in the treatment of eye diseases	Crushing fresh leaves and squeezing the juice	Asfaw et al. (2023)

In addition, Avicenna also described usage of nightshade in the treatment of various diseases, for example, an ointment from nightshade has been used for the treatment of headaches and different types of tumors (earlobes and meninges); also plant fruits juice has been used for eyes and throat diseases, and as a sleeping pill as well. Moreover, nightshade fruits were used as hemostatic and diuretic agents for excessive menstrual bleeding, and also for diseases of the kidneys and bladder as well. According to Avicenna “The Canon of Medicine,” nightshade also relieves pain (Khojimatov, 2021; Boboev et al., 2023).

Historically Central Asian botanists, especially in the 20th century, made significant contributions to developing pharmacognosy, pharmacology and phytotherapy (Khojimatov et al., 2020; 2023a). Sezik et al. (2004) worked on examining folk medicine in Uzbekistan, which resulted in 177 folk remedies in the surveyed area of Jizzakh, Samarqand, and Tashkent provinces. Among these folk remedies, 162 were obtained from 79 different plant species belonging to 31 families, including 15 animal-originated remedies belonging to eight animals (Khojimatov et al., 2020; 2023a). Nonetheless, almost no research has been done on the application of members of the Solanaceae family, particularly the species of the genus *Solanum*, in folk medicine in Uzbekistan. Recently, there is limited information available concerning the ethnobotanical use of three species, *S. lycopersicum*, *S. melongena* and *S. tuberosum* within the region of Uzbekistan (Solanum-Lycopersicum, 2023; Solanum-Melongena, 2023; Solanum-Tuberosum, 2023).

*Solanum* species have a long history of uses both as edible and as ethnomedicinal plants in different traditional practices around the world. The species have long been used in folk medicine to treat various illnesses, including constipation, eczema, hemorrhoids, heart diseases, herpes, inflammations, rheumatism, wounds, etc. (Kaunda and Zhang, 2019). Various parts of many species belonging to the *Solanum* section are widely used in medicine all over the world. Their use as such is recorded from the earliest times, and various species, especially *S. nigrum*, are mentioned and often illustrated in all of the ancient herbals, with Dioscorides being one of the first to record their medicinal properties. Since then, *S. nigrum* has continued to be widely acclaimed for its medicinal effects in every country where the taxon is found. The previous study demonstrates that the *Solanum* species, as the most famous member of the Solanaceae family, has noticeable traditional applications that mainly originate from South America, Asia, Africa and Europe. In American and Asian countries, particularly India, Brazil, Mexico, and China, there are special reports on the traditional applications of *Solanum* species. Besides *Solanum* in Solanaceae in Uzbekistan, Khojimatov et al. (2020) reported traditional uses of the two species from the Solanaceae family (*Datura stramonium* L. and *Hyoscyamus niger* L.).

Therefore, this study aimed to review the ethnobotanical knowledge of *Solanum* species (Table 2). Based on the available literature data, ethnographic, ethnopharmacological and food uses of the eight *Solanum* species from Uzbekistan are reported. Among them, *S. nigrum* is one of the largest, most variable/widespread species of the genus *Solanum* (Eskandari et al., 2019) and the most used in traditional medicine worldwide, while on the other hand, *S. dulcamara* and *S. melongena* are practically not documented. Among the selected *Solanum* species, the most literature data were based on *S. tuberosum* followed by *S. lycopersicum*, *S. nigrum*, *S. melongena*, *S. villosum, S. rostratum*, while *S. dulcamara* and *S. sisymbriifolium* were least examined and explored.

Chen et al. (2022) reviewed that the first known record describing the medicinal use of *S. nigrum* was found in Yao Xing Lun (药性论, Tang Dynasty) (Editorial Committee of State Administration of Traditional Chinese Medicine, 1999). Since then, its medicinal use was increasingly reported in many other well-known classical, traditional Chinese medicine (TCM) monographs, including Ben Cao Gang Mu (本草纲目, Ming Dynasty), Ben Cao Gang Mu Shi Yi (本草纲目拾遗, Qing Dynasty), Ben Cao Tu Jing (本草图经, Song Dynasty), Dian Nan Ben Cao (滇南本草, Ming Dynasty), Dian Nan Ben Cao Tu Shuo (滇南本草图说, Ming Dynasty), Jiu Huang Ben Cao (救荒本 草, Ming Dynasty), Shi Liao Ben Cao (食疗本草, Tang Dynasty), and Xin Xiu Ben Cao (新修本草, Tang Dynasty). In all of these major TCM monographs, it was recorded that *S. nigrum* has different medicinal properties and TCM herbs or classical prescriptions containing *S. nigrum* have been used as decoction, granules, pills, powders, and tablets (Chen et al., 2022). The traditional usages of *S. nigrum,* commonly used worldwide, are presented in Table 2. Melongianum (*S. melongena*) was domesticated in Vavilov’s Chinese center (Vavilov, 1992) or Indo-Chinese center (Vavilov, 1992) and was known in the Middle Ages as well (Carnevale Schianga, 2011). It is included in the *Tractatus de herbis* and other similar manuscripts (Touwaide and Appetiti, 2013).

## Conclusion

Species of the genus *Solanum* are considered either valuable foodstuffs and/or important medicinal plants due to their wide range of applications in the field of ethnobotany. The diversity of *Solanum* as food brings undeniable health benefits to the population through the presence of starch (source of sugar), lycopene and phenolics (antioxidant), anthocyanins possess antidiabetic, anticancer, anti-inflammatory, antimicrobial, and anti-obesity effects, as well as prevent cardiovascular diseases.

Generally, the diverse phytochemical profiles of these *Solanum* species, encompassing alkaloids, flavonoids, sterols, saponins, and various other bioactive compounds, underscore their potential contributions to medicine and nutrition.

In conclusion, the review paper highlights the ethnobotanical significance of eight *Solanum* species in Uzbekistan, revealing their economic, nutritional, and medicinal values. These species demonstrate a diverse range of traditional uses, exhibiting potential as antibacterial, antifungal, anti-inflammatory, anticancer, and antioxidant agents. Nevertheless, the research also highlights a decline in the transmission of traditional knowledge. This underscores the importance of ongoing phytochemical investigations to fully leverage the medicinal capabilities of *Solanum* species. The discoveries presented in this study offer valuable insights for future research and the development of innovative pharmaceutical solutions. They encourage the exploration of new plant sources and the utilization of advanced pharmaceutical methodologies in the pursuit of potential drug development.
