# Supplementary material for: Exploring biodiversity and ethnobotanical significance of Solanum species in Uzbekistan: unveiling the cultural wealth and ethnopharmacological uses
Source: Front Pharmacol. 2024 Jan 24;14:1287793. doi: 10.3389/fphar.2023.1287793 (PMC10851437; doi:10.3389/fphar.2023.1287793)
Supplement: Supplementary file 1 [file DataSheet1.PDF]

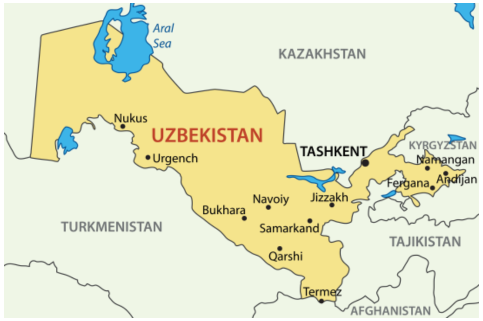

Species of *Solanum* in Uzbekistan

Diversity Habitat and ecology Phenology Population status Global distribution Reproduction Food

Description Cultivation Invasiveness Wild Application Human disease

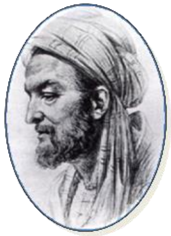

Avicenna
credit by **https://uz.wikipedia.org/wiki/Abu_Ali_ibn_Sino#/media/Fayl:Avicena.jpg**

Ethnobotanical study of the eight *Solanum* species

Ethnopharmacological uses of *Solanum* spp.

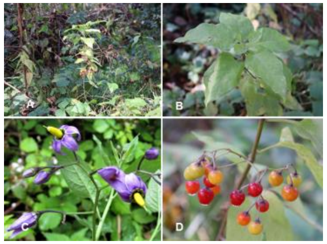

Solanum dulcamara

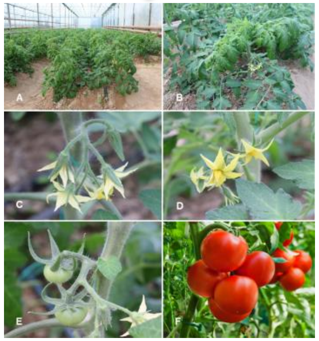

Solanum lycopersicum

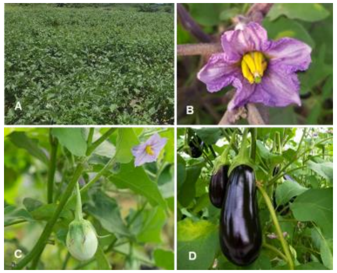

Solanum melongena

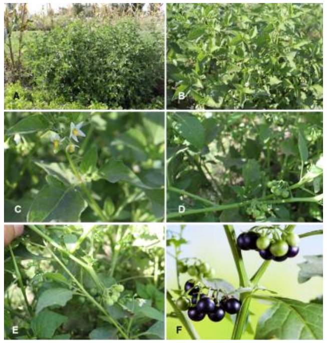

Solanum nigrum

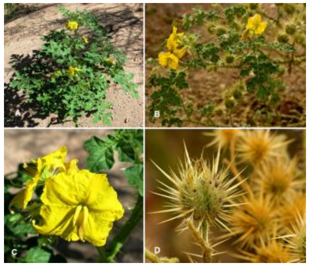

Solanum rostratum

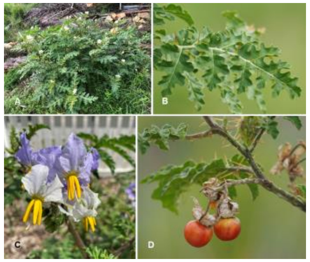

Solanum sisymbriifolium

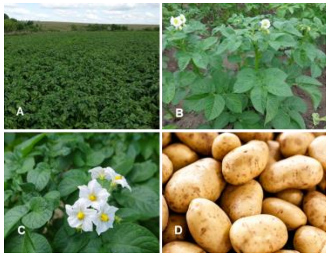

Solanum tuberosum

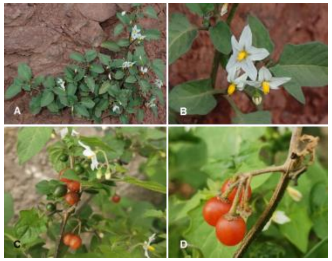

Solanum villosum
